# Advances in MALDI Mass Spectrometry Imaging Single Cell and Tissues

**DOI:** 10.3389/fchem.2021.782432

**Published:** 2022-02-03

**Authors:** Xiaoping Zhu, Tianyi Xu, Chen Peng, Shihua Wu

**Affiliations:** ^1^ Joint Research Centre for Engineering Biology, Zhejiang University-University of Edinburgh Institute, Zhejiang University, Haining, China; ^2^ Research Center of Siyuan Natural Pharmacy and Biotoxicology, College of Life Sciences, Zhejiang University, Hangzhou, China

**Keywords:** matrix-assisted laser desorption/ionization (MALDI), imaging mass spectrometry, single-cell metabolomics, proteomics, spatial distribution, tissue mapping

## Abstract

Compared with conventional optical microscopy techniques, mass spectrometry imaging (MSI) or imaging mass spectrometry (IMS) is a powerful, label-free analytical technique, which can sensitively and simultaneously detect, quantify, and map hundreds of biomolecules, such as peptides, proteins, lipid, and other organic compounds in cells and tissues. So far, although several soft ionization techniques, such as desorption electrospray ionization (DESI) and secondary ion mass spectrometry (SIMS) have been used for imaging biomolecules, matrix-assisted laser desorption/ionization (MALDI) is still the most widespread MSI scanning method. Here, we aim to provide a comprehensive review of MALDI-MSI with an emphasis on its advances of the instrumentation, methods, application, and future directions in single cell and biological tissues.

## 1 Introduction

Mass spectrometry (MS) is a fundamental analytical technique for sensitive detection and identification of hundreds of inorganic elements and organic molecules in complex mixtures. Since 1912, J.J. Thomson found that isotopes of neon had masses 20 and 22 in a 10:1 ratio and explained its apparently anomalous atomic weight of 20.2. MS became more and more important for many life and science fields. A large number of MS methods and instruments, including ion sources, detectors, and analyzers have been developed (Wiseman et al., 2009). In the last three decades, with the advent of soft ionization techniques, such as electrospray ionization (ESI) (Fenn et al., 1989) and matrix-assisted laser desorption/ionization (MALDI) (Karas et al., 1987; Tanaka et al., 1987), it became possible to obtain mass spectra of proteins, DNA/RNA, carbohydrates, lipids, polymers, etc. In addition, with dramatic instrument improvements in unique capabilities of specificity, sensitivity, speed, sampling, and automated computer data acquisition/reduction, MS became an indispensable tool for the label-free detection of intact biomolecules in biological samples.

**FIGURE 1:**
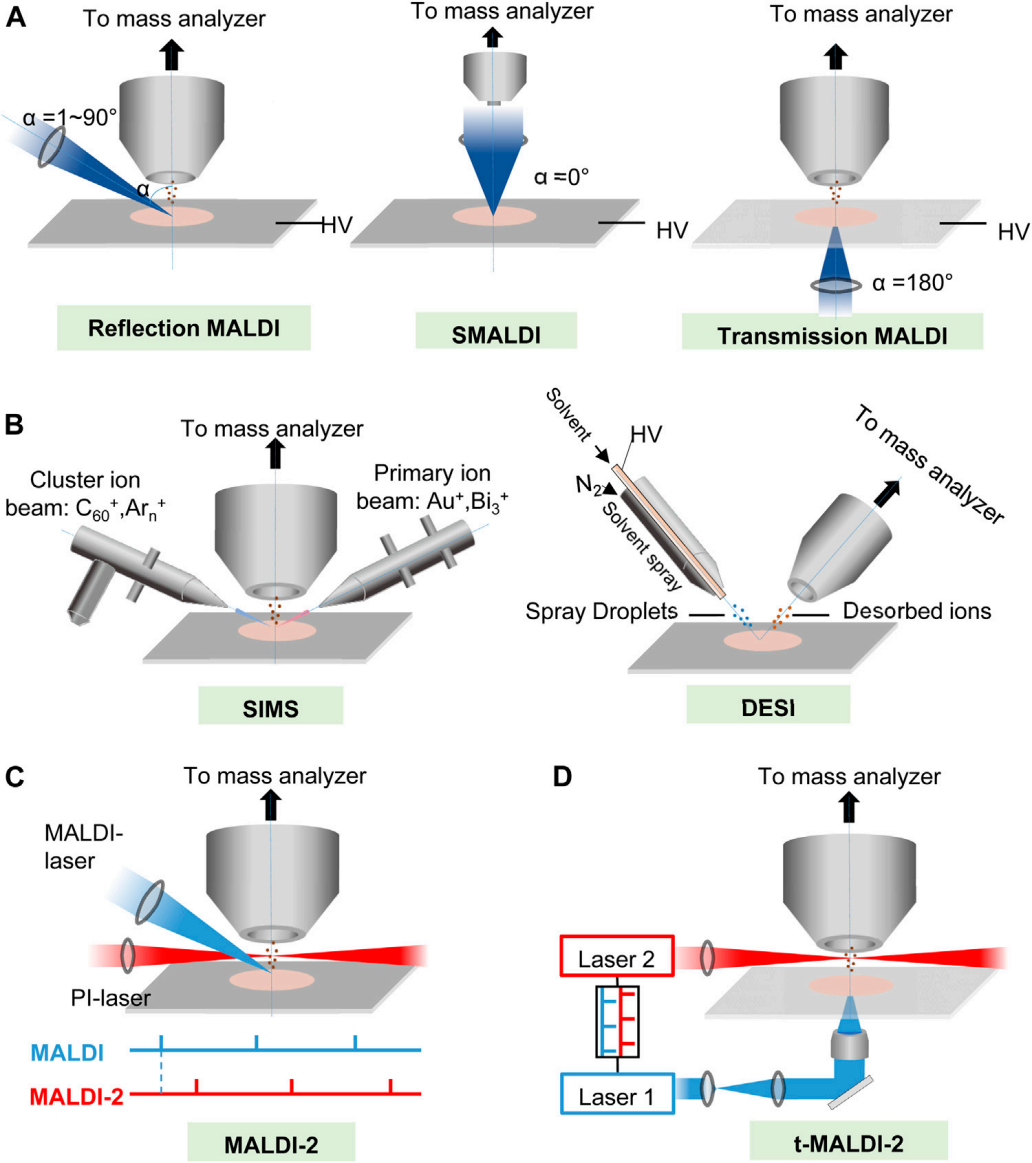
The principles of **(A,B)** major MSI ionization methods and **(C,D)** two kinds of new post ionization (PI) laser enhanced new high-resolution MALDI ion sources. **(A)** Schematic of MALDI at several different laser angles (α). HV, high voltage. **(B)** Schematic of SIMS and DESI. **(C)** Schematic drawing of the modified MALDI ion source of the Synapt G2-S mass spectrometer. Primary MALDI and PI laser beams for more complete ionization and shielding aperture for increasing the cooling gas pressure in the region of ion generation. The lower panel illustrates the laser pulse triggering sequence. **(D)** Schematics of t-MALDI-2–MSI. An actively Q-switched Nd: YLF laser (laser 1, λ = 349 nm, M2 ∼ 1.05) is focused onto a matrix-coated sample via a UV-transmitting ×50 objective in back-side illumination geometry. The Nd: YAG laser (laser 2 wavelength 2,266 nm) intersects the extended analyte matrix plume at a certain distance and a certain delay between the two laser pulses.

Meanwhile, the development of approaches for detecting, identifying, and mapping spatially the localization of molecules using mass spectrometry imaging (MSI, also named as imaging mass spectrometry) has extended these strengths of analytical MS to the cellular and subcellular scales and enabled detailed molecular mapping of hundreds of molecules in biological tissues (Unsihuay et al., 2021a). MSI can provide detailed maps of hundreds of molecules in complex samples with high sensitivity and subcellular spatial resolution. As shown in Figures 1A,B and Table 1, up to now, there are several MSI ionization methods, such as desorption electrospray ionization (DESI), matrix-assisted laser desorption/ionization (MALDI), and secondary ion mass spectrometry (SIMS) (Gilmore et al., 2019; Schnackenberg et al., 2021). Similar to high-resolution MALDI ion sources, secondary ion mass spectrometry (SIMS, Figure 1B) ion beam technology has become a complementary mainstream method from the fringe of biological imaging due to significant developments in the primary ion beam technologies and mass spectrometers (Passarelli et al., 2017; Gilmore et al., 2019; Newell et al., 2020; Samfors and Fletcher, 2020; Sparvero et al., 2021; Van Nuffel et al., 2021). However, due to the ease of sample preparation, user-friendliness, speed, high sensitivity, and easy-to-interpret spectra, MALDI is still one of the most suitable MS ionization techniques for MSI in clinical laboratory (Norris and Caprioli, 2013a; Flatley et al., 2014; Schulz et al., 2019; Schnackenberg et al., 2021).

**TABLE 1:** Differences between matrix-assisted laser desorption/ionization (MALDI), secondary ion mass spectrometry (SIMS), and desorption electrospray ionization (DESI) (Svatos, 2010; Yang et al., 2020).

	MALDI	SIMS	DESI
Beam source	Primary ion	Secondary ion	Primary ion
Ionization method	UV-laser	primary charged particles (Cs^+^, SF_5_^+^, Au, and Sb clusters, C_60_^+^) eject and ionize material from surface	Modified ESI source spraying solvent using high-pressure gas flow on the sample surface
Sample preparation	Freezing microtome section and matrix application is needed	Freezing microtome section is needed, then directly analyzed	Directly analyzed
Matrix	Needed	None	None
Environment	Vacuum	Vacuum/low pressure	Atmosphere
Space resolution	10–100 µm	100 nm∼1 µm	40–200 µm
Sample damaging level	High	Low	Low
Detected object	All kinds of biological samples, no limitation of molecular weight	Hydrophobic compounds with molecular weight not exceeding 1,000 Da	Compounds with molecular weight not exceeding 2,000 Da

MALDI-MSI is a label-free, innovative, and emerging technique that produces two-dimensional (2D) ion density maps representing the distribution of an analyte(s) across a tissue section in relation to tissue histopathology. One main advantage of MALDI-MSI over other imaging modalities is its ability to determine the spatial distribution of hundreds of analytes within a single imaging run, without the need for a label or any prior knowledge (Schnackenberg et al., 2021). Furthermore, MALDI produces mainly singly charged ions, providing a less complex analyte ion profile than ESI where the occurrence of multiple charged ions from the same analyte tends to crowd the spectrum and renders interpretation difficult (Flatley et al., 2014). Over the last decade, MALDI-MS imaging has been used by researchers to explore areas of proteomics, lipidomics, and metabolomics in biological and clinical samples (Norris and Caprioli, 2013a; Chen et al., 2015; Cole and Clench, 2015; Rocha et al., 2017; Capitoli et al., 2019; Pareek et al., 2020; Schnackenberg et al., 2021). Histology-directed MS measurements provide unique insight into clinical questions for histology images and help to diagnose disease. MALDI-MS imaging, underpinning with enough specific molecular information, is poised to revolutionize the practice of anatomic pathology in the coming decade, and provide the next generation of diagnostic tests that will extend and improve the quality of life (Norris and Caprioli, 2013b). Although there are numbers of advances and applications in the versatile fields, in this review, we will highlight the recent key advances and application of MALDI-MSI with an emphasis on the instrumentation, methods, application, and future directions in single cell and biological tissues.

## 2 Matrix-Assisted Laser Desorption/Ionization Ion Sources and Mass Analyzer

The most basic MALDI process is to mix a concentrated matrix solution with an analyte solution and then dry it on a MALDI target plate to produce a matrix/analyte crystalline spot. Due to the high molar excess of the matrix, the matrix-dominated sample crystals absorb pulsed laser energy, leading to desorption and ionization of the matrix/analyte through the sample volume disintegration process (Cornett et al., 2007; Flatley et al., 2014). The information content of MS images is critically influenced by a combination of the 1) laser focusing optics/geometry, 2) precision of the sample positioning stage, 3) source pressure, 4) ion transfer, and 5) capabilities of the mass analyzer. (Roempp and Spengler, 2013; Gilmore et al., 2019). The ideal mass spherometer for MALDI-MSI would satisfy the “4S-criteia for performance” (speed, specificity, spatial resolution, and sensitivity). Each of these will impose limitations on the achievable lateral resolution and the information obtained from acquired mass spectra.

### 2.1 Classical Ion Sources

Historically, MALDI technology was first developed by Koichi Tanaka when he used the “metal fine powder and glycerol matrix” method for the same preparation (Tanaka et al., 1987). This method made a breakthrough of low molecular weight limit of laser desorption time-of-flight mass spectrometry (LD-TOF-MS). At that time, May 1987, the mass number that we had been able to measure had already exceeded 48,000 Da. Soon after this, the measured mass numbers were extended in the range of 72,000 to 100,000 Da (Tanaka et al., 1988). Almost at the same time as Tanaka, Karas and Hillenkamp also developed the MALDI method (Karas et al., 1987) to measure proteins with molecular masses exceeding 10,000 Da in 1987 (Karas and Hillenkamp, 1988). The novel soft ionization technique could introduce larger biomolecules such as proteins into the mass spectrometer (Flatley et al., 2014) and, thus, won dramatic applications and improvements. After 10 years, in 1997, MALDI was applied to biological imaging biological samples, e.g., human buccal mucosa cells and endogenous proteins (Caprioli et al., 1997). This group then went on to automate the MALDI-MSI process and demonstrated the application of the technique to imaging proteins and peptides in the mouse brain and glioblastoma sections. Soon, the application areas have rapidly expanded to include a large number of small molecules, e.g., small-molecule drugs, peptides, lipids, and neurotransmitters (Trim and Snel, 2016).

So far, there are several kinds of MALDI methods to generate ions. As shown in Figure 1A, according to the angle (*α*) of laser irradiation with respect to the sample surface normal and the pressure in the ion source, MALDI-MSI systems may be grouped into three major categories, transmission and reflection MALDI, and scanning microprobe matrix-assisted laser desorption/ionization (SMALDI) (Gilmore et al., 2019). For a classical transmission MALDI, the laser beam irradiates the sample at 180° relative to the analyzer axis; thus, the focused laser beam is required to penetrate the sample, and ions are ejected in the direction of laser propagation. The transmission geometry has been successfully employed for high lateral resolution lipid/metabolite MALDI-MSI with pixel resolutions down to 5 μm. While for the reflection of MALDI, the laser beam irradiates the matrix-covered sample surface with an angle between 0° and 90° regarding the analyzer inlet, which means ions are “reflected” from the sample surface, and sample perforation is not required. A previous study (Potocnik et al., 2015) indicated that using the rapifleX MALDI-TOF-MSI instrument to image lipid distributions in tissue sections, it was found that lateral resolutions were down to 10 μm. Scanning microprobe matrix-assisted laser desorption ionization (SMALDI) mass was invented by Spengler, B. and Hubert, M. in 2002 (Spengler and Hubert, 2002). It was further improved as atmospheric pressure scanning microprobe matrix-assisted laser desorption/ionization mass spectrometry (AP-SMALDI MS) for imaging (Vegvari et al., 2017; Bhandari et al., 2018; Bredehoeft et al., 2019; Kadesch et al., 2020; Mokosch et al., 2021; Mueller et al., 2021; Righetti et al., 2021), while atmospheric pressure MALDI ion sources AP-MALDI have also been developed for MSI (Guenther et al., 2011; Jackson et al., 2018; Keller et al., 2018) and achieved a lateral resolution at 1.4 μm (Kompauer et al., 2017).

### 2.2 New Post-Ionization Laser-Enhanced Ion Sources

As described above, MALDI-MSI can simultaneously record the lateral distribution of numerous biomolecules in tissue slices, but its sensitivity is restricted by limited ionization. Recently, Jens Soltwisch et al. (Soltwisch et al., 2015) introduced a wavelength-tunable PI laser strategy, called MALDI-2 (Figure 1C), that initiates secondary MALDI-like ionization processes in the gas phase. In MALDI-2, the beam of a pulsed ultraviolet (UV) laser intercepts the expanding particle plume in an N_2_ cooling gas environment, which contrasts with previous photoionization studies where classical high-vacuum ion sources (*p* ≤ 10^–6^ mbar) were implemented. An effective diameter of ∼5 μm of the primary laser beam was achieved by beam shaping and by mounting the focusing lens inside the MALDI ion source. In this way, the ion yields for numerous lipid classes, liposoluble vitamins, and saccharides could be increased, and imaged in animal and plant tissue with a 5-μm-wide laser spot, by up to two orders of magnitude. Critical parameters for initiation of the secondary ionization processes are pressure of the cooling gas in the ion source, laser wavelength, pulse energy, and delay between the two laser pulses. The technology could enable sensitive MALDI-MS imaging with a lateral resolution in the low micrometer range (Soltwisch et al., 2015; Soltwisch et al., 2020; Bien et al., 2021b).

The mechanisms underlying ionization by proton transfer in MALDI-2 possibly involve resonant two-photon ionization of the matrix (m) by the PI laser (giving rise to m^+•^ ions and free e^–^), succeeding collisions with neutral matrix molecules (leading to the generation of protonated or deprotonated matrix), and proton transfer to or from neutral analyte molecules (M) in subsequent collisions to yield the observed [M + H]^+^ or [M—H]^–^ products (Soltwisch et al., 2015). Thus, with the use of optimal PI laser conditions, increased ion signals were produced. With the idea, and with the use of laser-induced post-ionization transmission-mode MALDI ion sources (named t-MALDI-2, Figure 1D) and an Orbitrap mass analyzer to compose a t-MALDI-2 MSI system, M. Niehaus et al. (Niehaus et al., 2019) achieved a high a pixel size of 600 nm with brain tissue. The method could constitute a valuable new tool for research in cell biology and biomedicine.

### 2.3 Mass Analyzer

MALDI has been used in combination with TOF mass spectrometer for MALDI MSI, which could provide high spatial resolution (10 μm and better) and fast acquisition speed (e.g., provided by a 10-kHz laser), but they are less suitable for small molecules. Fourier Transform (FT) ion cyclotron resonance (ICR) or FT-orbitrap mass analyzer high resolving power and mass accuracy can provide high resolving power and mass accuracy, which are key for small molecule MSI and determinants of specificity. Currently, FT-ICR MS could provide the highest resolving power (>1,000,000 at *m/z* 200) capable of resolving isotopic fine structure. MALDI Orbitrap FT-MS also can provide high resolving power (>140,000) and lateral resolution. Some MALDI-QTOF instruments with intermediate resolving power combine MSI with ion mobility separation as a complementary separation technique that offers the potential to separation isometric molecules (Schulz et al., 2019).

## 3 General Process of MALDI-MSI

**FIGURE 2:**
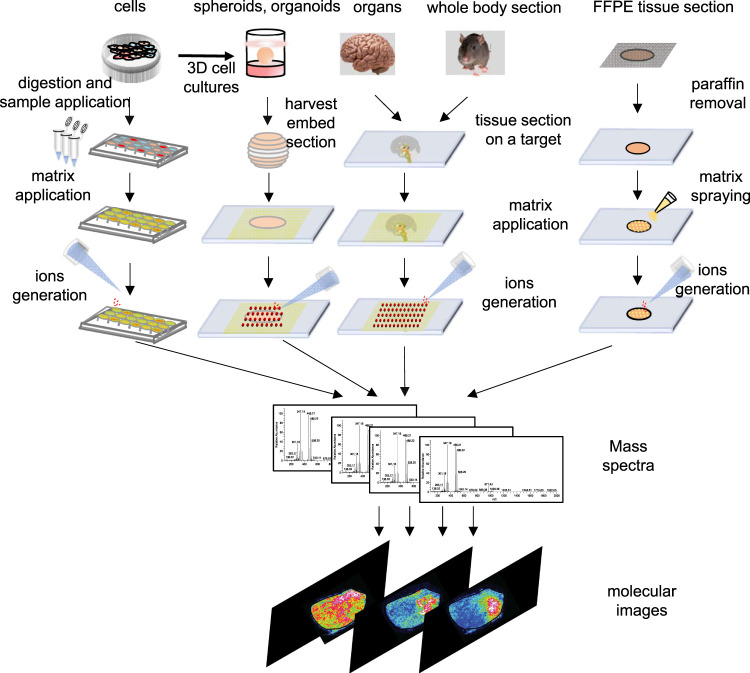
Schematic outline of workflow of cells, spheroids, organ and tissue section, whole-body section and formalin-fix, paraffin-embedded (FFPE) tissue section for an MSI experiment in different samples.

Principally, MALDI-MSI operation is a very simple and convenient process. As shown in Figure 2, the MSI sample preparation method includes sample collection, storage, sectioning, tissue pretreatment, matrix spraying, and so on, and is related to the type of sample and the nature of the object to be detected. Usually, a concentrated matrix solution is first mixed with the analyte solution and allowed to dry on a MALDI target plate (Flatley et al., 2014). After sufficient dry matrix coverage is achieved, the sample with high molar excess of the matrix is then recrystallized (Yang and Caprioli, 2011). The recrystallization allows analyte extraction from the sample surface in a controlled manner without a washing or spraying motion, leading to matrix/analyte desorption and ionization by a sample volume disintegration process (Yang and Caprioli, 2011). Sublimation and recrystallization offers the best spatial resolution in terms of crystal size, which allows the laser spot of the MALDI to define the spatial resolution of the experiment (Bouschen et al., 2010).

In the sample preparation process, even a slight movement in the analytes from their native positions is amplified. Thus, the main difficulty in the preparation of MALDI-MSI experimental tissue is that the chemical integrity of the targeted metabolites is not changed, and the true spatial distribution of the metabolites in the tissue is retained to minimize the displacement (Sturtevant et al., 2021). What is more, avoiding large volumes of matrix solvent is critical to maintaining optimal spatial resolution when performing MSI on small samples (Weaver and Hummon, 2013).

### 3.1 Sample Types and Pretreatment

MSI enables local molecular analysis at a broad range of length scales, from the subcellular level to whole body tissue sections, including organ tissue section, whole body section, spheroids in 3D cell cultures, and formalin-fixed and paraffin-embedded (FFPE) tissues, and their sample preparation processes are different.

#### 3.1.1 Cell Sample

Cell samples from 2D monolayer cultures can also apply to MALDI-MSI. The sample preparation procedure is depicted in Figure 2. First, cells cultured in cell culture flasks are digested with trypsin. The cells are centrifuged, and the supernatant is discarded. The cell pellet is subsequently washed twice with PBS solution before re-suspended in PBS to obtain a cell suspension. Cell suspensions are deposited onto the MALDI target. After drying of the cell suspension liquid, the matrix is dropped onto the analyte and then used for MALDI-MSI (Chen et al., 2017).

Recently, Tanja Bien et al. demonstrated the pros and cons of the protocols with four model cell lines, cultured directly on indium tin oxide (ITO)-coated glass slides, and achieved the cultures at a pixel size of 2 μm by using transmission (t-) mode MALDI-2-MSI enabled on an Q Exactive plus Orbitrap mass spectrometer (Bien et al., 2021a). Cells were cultured directly on ITO-coated glass slides (70–100 Ω/sq, Merck) equipped with growth chambers. A total number of 2 × 104 cells in 0.5 ml of growth media were directly sown into each chamber of the slides and grown for 48 h to subconfluence. It should be noted that the direct growth on the ITO surface results in a slightly reduced adherence of the cells compared with the original glass slide of the chamber slide assembly.

#### 3.1.2 Spheroid 3D Cell Cultures

Spheroids and organoids are three-dimensional (3D) cell models when cultured in suspension or nonadhesive environment, which is different from 2D monolayer cultures. Organoids are *in vitro* models of human development and disease study. They are often thought of as miniature versions of organs, and they often show very precise microscopic anatomical structures. Spheroids are 3D cultures composed of cellular polymers produced by a single cell type or a mixture of cells. Nowadays, 3D cell culture is widely used in screening environments for better assessment of drug safety and identifying potential cancer therapeutics (Lin and Chang, 2008; Hirschhaeuser et al., 2010).

In 2011, MALDI-MSI was combined with 3D cell culture to examine protein distribution for the first time, and it was found that cytochrome C and Histone H4 are the two predominant proteins in the 3D colon carcinoma cultures (Li and Hummon, 2011). Later, further studies of 3D cell culture about colon carcinoma (Liu et al., 2018c), human skin (Avery et al., 2011; Russo et al., 2018), blood–brain barrier (Bergmann et al., 2018), pancreatic cancer (Johnson et al., 2020), colorectal cancer (Liu et al., 2018b), and breast cancer (David et al., 2018) are combined with MALDI-MSI to analyze metabolites. Besides, Flint et al. characterized the metabolites, proteins, and metals of a novel aggregated spheroid model, termed “aggregoid,” by DESI-MSI, and they demonstrated that absolute quantification of drugs is achievable in 3D tissue models (Flint et al., 2020). It is obvious that the combination of MSI and 3D cell culture has become a promising tool for early-stage drug analysis and disease analysis.

The basic workflow of 3D cell models used in MSI is depicted in Figure 2. Cells are seeded and incubated with a layer of agarose dissolved in cell culture media at the bottom of the inner plates to facilitate 3D spheroid and organoid formation. After 10–14 days, the spheroids grow to roughly 1 mm in diameter. The 3D cell models are then collected and embedded in gelatin for sectioning and subsequent MSI analysis (Weaver and Hummon, 2013). Details about sample preparation strategies for MSI of spheroid 3D culture models can be found in Wheatcraft et al. (2014) and sample preparation strategies for MSI of primary tumor organoids can be found in Johnson et al. (2020).

#### 3.1.3 Organ Tissue Sections

Fresh frozen tissue slices (5–15 μm) collected from organs and whole body are the most common samples used in MALDI-MSI. Fresh frozen tissues, which are cut on a cryostat, are thaw mounted on a metal target or conductive glass slide. Excess lipids and salts can interfere with matrix crystallization and analyte ionization when analyzed protein, endogenous soluble ionization-suppressing compounds, and salts can interfere the detection of small molecules; therefore, sections are necessary to be washed by organic solvents (Lemaire et al., 2006; Schwamborn and Caprioli, 2010; Yang and Caprioli, 2011; Shariatgorji et al., 2012; Wheatcraft et al., 2014; Sun et al., 2019). The major process for the preparation of fresh frozen tissue sections for direct analysis by MALDI-MS is summarized in Figure 2 (Schwartz et al., 2003; Goodwin, 2012).

Histologically, tissues are often stained for observation. Some histological stains, such as cresyl violet or methylene blue, are compatible with subsequent MS analysis (Chaurand et al., 2004). However, other stains, such as hematoxylin and eosin (H&E), can interfere subsequent MS analysis; therefore, serial sections are obtained and stained to guide matrix deposition and laser ablation and to allow comparison of MS results with tissue histology (Schwamborn and Caprioli, 2010). Another solution is performing MS analysis first and staining after removing the matrix (Grey et al., 2009).

#### 3.1.4 Formalin-Fixed and Paraffin-Embedded Tissues

In contrast to fresh frozen tissue, FFPE tissue specimens are the well-established processing methods employed in histological examination. They are prepared by immersing the sample in formalin to fixation, then removing the fixator and residual water in ethanol and using an organic solvent (such as xylene) to remove the ethanol, and finally embedding the sample in molten paraffin. Thus, FFPE samples have been through to be unusable for proteomic approaches because of protein cross-linking caused by formalin fixation for a long time (Becker et al., 2007). In 2007, the I. Fournier’s group presented two methods for direct analysis of FFPE tissues by MALDI-MS (Lemaire et al., 2007), making it possible to get massive amounts of archived samples in the clinical pathology setting. In addition, the use of antigen retrieval techniques and *in situ* tryptic digestion has allowed the analysis of FFPE samples by MSI (Groseclose et al., 2008).

Recently, a MALDI-MSI protocol for tryptic peptides from FFPE tissues (Ly et al., 2019), which help to establish a standard operating procedure. High-mass-resolution MALDI-FT-ICR-MSI platform had also been used for the *in situ* analysis of metabolite content from the FFPE sample (Ly et al., 2016). Using this platform, an overlap of 72% of detected species was achieved in the mass range of *m/z* 50–1,000 in FFPE samples, compared with fresh frozen samples. Metabolites are found to be largely conserved in FFPE tissue samples, and thus, the data acquired with this protocol can be used in research and clinical practice, making full use to mining data in traditional FFPE tissue. Recent research applied MALDI-MS to FFPE tumor tissue sections and enabled cancer subtype classification, providing a promising complementary approach to current pathological technologies for precise digitized diagnosis of diseases (Möginger et al., 2020).

Nowadays, MSI is a powerful tool that has been used to detect biomarkers, such as peptides (Groseclose et al., 2008), proteins (Lemaire et al., 2007; Stauber et al., 2008; Araujo et al., 2014), lipids (Carter et al., 2011; Denti et al., 2020), metabolites (Djidja et al., 2017; Clift et al., 2021), and N-linked glycans (Bai et al., 1994; Eshghi et al., 2014; Gustafsson et al., 2015; Briggs et al., 2017) in FFPE tissues. Generally speaking, sample preparation of FFPE tissue applied in MALDI-MSI is slightly different from fresh frozen tissue (depicted in Figure 2 with additional steps to remove the paraffin). Micro-digestion is needed before matrix spraying in case of protein analysis.

#### 3.1.5 Whole-Body Sections

Whole-body autoradiography is a traditional technology carried out in animal tissues during the early stage of drug and metabolite distribution studies. However, there are a number of limitations about whole-body autoradiograph, especially the expensive synthesis of radiolabeled drugs and analyte specificity and identification.

MALDI-MSI has been shown to be more advantageous for imaging the distribution of drugs and metabolites in a whole-body section (Trim et al., 2008). In 2005, Rohner et al. performed MALDI-MSI and study the drug distribution in a whole-body mouse section for the first time (Rohner et al., 2005). Soon, whole body MALDI-MSI will be extended to detect proteins in a whole-body scale (Khatib-Shahidi et al., 2006). A typical MALDI-MSI process for whole-body tissue sections contained several steps. In brief, animals were deep frozen and embedded in precooled semiliquid gel of carboxymethylcellulose (CMC) and cut on a cryomicrotome. Then the sections were placed on a copper block and transferred to a desiccator with a membrane pump for section drying. The sections were mounted on metal plates using double-side adhesive tape and followed by matrix coating and MALDI-MSI. Considering the size of the sample, whole-body MALDI-MSI is usually used to study some small animals such as nematodes (Hameed et al., 2015), fruit flies (Khalil et al., 2017), and mice (Huber et al., 2018; Saigusa et al., 2019).

### 3.2 Standards Addition and Quantification

**FIGURE 3:**
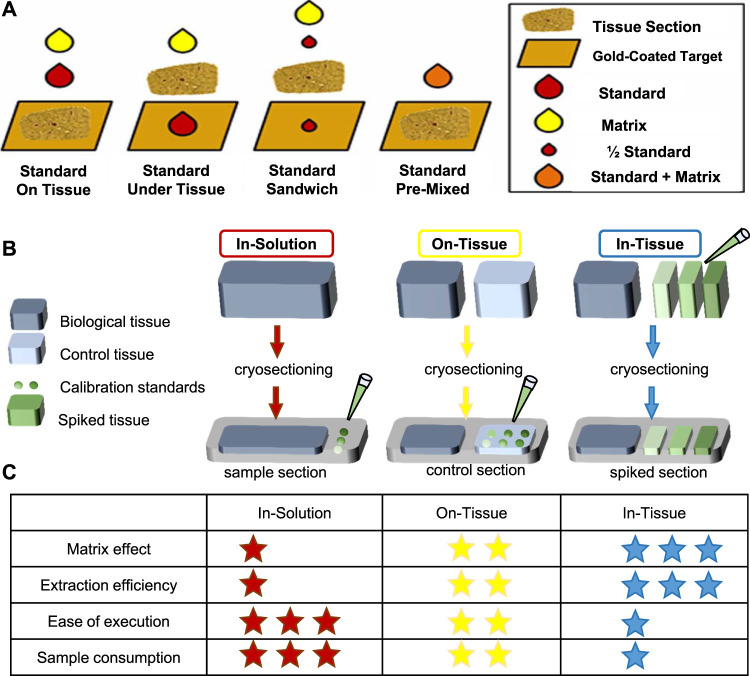
Representative standard applyed methods. **(A)** Four methods of applied internal standards for MSI (Chumbley et al., 2016). **(B, C)** Three common methods for calibration of standards applied. Modified from Porta et al. (2015) and Tobias and Hummon (2020). **(B)** The in-solution method is to directly spot the calibration standard on the indium tin oxide (ITO) slide. On the other hand, the on-tissue method places a control sample next to the sample section and spot calibration standards on the control section. In addition, the in-tissue method uses a tissue simulation model spiked with calibration standards of different concentrations, and the tissue simulation model is section and placed next to the sample portion. **(C)** A summary table of the characteristics of each method, where the asterisk indicates performance (low = 1 star, high = 3 stars).

Absolute quantification is one of the key challenges in the MS analysis of complex mixtures. Aside from instrumental parameters, analyte recovery from tissue and ionization matrix effects are critical for quantitative MSI on tissue sections (Porta et al., 2015). Interactions between analyte and the tissue may result in different analyte recoveries. Besides, the absence of chromatographic separation enhances the effect of ionization competition and variation in ionization efficiencies (Stern et al., 2017), and the uniformity of matrix deposition can also affect ionization (Tobias and Hummon, 2020). Thus, isotopically labeled internal standards are used; specific preparation of calibration standard is critical for quantification in MALDI-MSI. There are generally four methods of applying internal standards on tissue for MSI depicted in Figure 3A, including standard on tissue, standard under tissue, standard sandwich, and standard premixed. However, depositing the standards on the tissue followed by the matrix was found to be the most accurate for quantitative MALDI-MSI (Chumbley et al., 2016; Unsihuay et al., 2021a).

In addition, calibration curve is needed for absolute quantification, so application of calibration standards before internal standard and matrix is needed to create the calibration curve. There are three strategies of calibration standards applied—*in-solution* strategy, *on-tissue* strategy, and *in-tissue* strategy to be developed for quantitative MSI. Their advantages are summarized in Figures 3B,C. Other details about the construction of calibration curves especially obtaining the analytical figures of merit in qMSI are shared by Tobias et al. (Tobias and Hummon, 2020).

### 3.3 Matrix Application

Coating matrix on the sample plate is a key step in MALDI-MSI analysis. It is possible that the matrix of MALDI puts considerable pressure on the spatial integrity of general biological samples, especially single-cell samples (Schober et al., 2012), which results in cellular components that may be broken and leaked. Fortunately, choosing a suitable matrix and suitable deposition method can avoid loss of analytes (Baker et al., 2017). In addition, the use of optimized matrix can promote the effective ionization of the target analyte and generate uniform and small eutectic, which is necessary for obtaining high spatial resolution images (Vallianatou et al., 2019). The ideal matrix generally has the following properties: strong electron absorption at the adopted laser wavelength, better vacuum stability, lower vapor pressure, and better miscibility with the analyte in the solid state (Calvano et al., 2018).

The choice of matrix can change the ionizable molecular weight and molecular species of the mass spectrometer. Up to now, there are numbers of matrices to be used for MALDI MS. Table 2 selected some popular MALDI matrices including organic small molecules, inorganic nanomaterials, reactive matrices (derivatization solutions), and ionic liquid matrix.

**TABLE 2:** Common matrices used for MALDI MSI targets (Tholey and Heinzle, 2006; Tobias and Hummon, 2020; Schnackenberg et al., 2021).

Matrix class	Matrix names	Targets
Classical organics	2,5-Dihydroxybenzoic acid (DHB)	Lipids, peptides, neuropeptides, drugs, small proteins
	α-Cyano-4-hydroxy cinnamic acid (CHCA/CCA)	Proteins, peptides, N-glycans, lipids
	Sinapinic acid (SA)	Proteins and peptides
	4-Chloro-α-cyanocinnamic acid (CICCA)	Proteins and peptides
	2,5-Dihydroxyacetophenone (2,5-DHAP)	Phospholipids, proteins
	9-Aminoacridine (9-AA)	Free fatty acids, lipids
	1,5-Diaminonaphthalene (1,5-DAN)	Glycolipids, metabolites
	2-(2-Aminoethylamino)-5-nitropyridine	Phospholipids
	2-Mercaptobenzothiazole	Phospholipids
	4-Nitroaniline (PNA)	Phosphatidylethanolamine
	Norhamane	Bile acids, lipids
	Dithranol	Di-and triacylglycerols
	1,6-Diphenyl-1,3,5-hexatriene (DPH)	Free fatty acids
	1,8-Bis(dimethylamino) naphthalene (DMAN)	Free fatty acids
	N1,N4-Dihbenzylidenebene-1,4-diamine (DBDA)	Fatty acids
	Meso-tetratkis (pentafluorophenyl)-porphyrin	Free fatty acids
	2,4-Dihydroxyacetophenone (DHAP)	Glycoproteins
	2.4,6-Trihydroxyacetophenone (THAP)	Lipids
	Picolinic acid	Oligonucleotides
	Succinic acid	Oligonucleotides
Reactive matrices	2.4-Diphenyl-pyranylium tetrafluoroborate (DPP-TFB)	Small molecule amines, neurotransmitters
	2.4,6-Trimethyl-pyranylium tetrafluoroborate (TMP-TFB)	Dopamine
	p-N,N,N-Trimethy lammonioanilyl N-hydroxysuccinimidyl carbamate iodide (TAHS)	Steroids and catecholamine
	4-Hydroxy-3-methoxycinnamaldehyde (CA)	
	2,3,4,5-Tetrakis (31,4-dihydroxylphenyl)thiophene (DHPT)	
	2-Fluoro-1-methyl pyridinium (FMP) derivatives	Neurotransmitters
Inorganic nanomaterials	Metal based (e.g., gold, silver, titanium oxide)	Small molecules
	Silicon based (e.g., nanopost arrays, nanowires, nanopillars)	Small molecules
Room-temperature ionic liquids	DHB-Py, DHB-MI (1-methylimidazole), DHB-TBA, SA-TBA	Small molecules
	CCA-DEA (N,N-diethylaniline), CCA-ANI (Aniline)	Peptides
	SA-TBA, SA- Et_3_N (triethylamine)	Proteins
	9-AA-NEDC	Lipids
	DHB-BuA (n-butylamine), CCA-MI, DHB-Py	Carbohydrates
	CCA-Py, CCA-MI, CCA-BuA	Phospholipids
	HPA (hydroxypicolinic acid)-DEA, CCA-ANI, CCA-MI	Oligonucleotides

#### 3.3.1 Common Matrix

Some matrices are popular due to their wide applicability, including α-cyano-4-hydroxycinnamic acid (CHCA) and 2,5-dihydroxybenzene. Formic acid (DHB) is used for peptides and metabolites (Tobias and Hummon, 2020). Different MALDI matrices have different sensitivities to different kinds of biomolecules. Perry et al. compared the intensity of various lipids in mouse liver tissue measured by the use of 9-aminoacridine (9AA), 5-chloro-2-mercaptobenzothiazole (CMBT), 1,5-diaminonaphthalene (DAN), and 2,5-dihydroxyacetophenone (DHA) and 2,5-dihydroxybenzoic acid (DHB), which provides a more reliable basis for the selection of MSI matrix (Perry et al., 2020). Liu et al. used n-phenyl-2-naphthylamine (PNA) with strong ultraviolet absorption and salt tolerance as a new type of matrix, which performed well in the analysis of a variety of small molecular metabolites including free fatty acids, amino acids, peptides, etc., then used for small-molecule imaging of rat middle cerebral artery occlusion (MCAO) brain tissue (Liu H. et al., 2018).

#### 3.3.2 Reactive Matrices for Chemical Derivatization

As described above, matrices, such as DHB and CHCA, may enhance analytical sensitivity. But for some specific molecules, such as small molecules or compounds with low ionization efficiency or in low abundance, there is still no suitable matrix, and thus, they will not be detected by the conventional MSI workflow. The signal peaks generated by organic matrices can also greatly interfere with the analysis of small molecules in MSI. Chemical derivatization makes it possible to perform targeted mass spectrometry imaging of these molecules (Tobias and Hummon, 2020). Derivatization solutions or reactive matrix serve as chemical matrices for laser desorption/ionization, can also enhance the detection of target molecules that are low in abundance, or contain certain chemical moieties (such as double bonds in amines or lipid fatty acyl groups) by reacting with them (Waldchen et al., 2019).

The on-tissue chemical derivatization (OTCD), which is spray chemical derivatization first followed by matrix application (Harkin et al., 2021), has been used to improve ionization efficiency to effectively detect analytes directly from both fresh frozen tissue and FFPE tissue. Now OTCD has developed rapidly and used to detect many biological molecules, such as N-glycan (Nishikaze et al., 2013; Holst et al., 2016; Zhang et al., 2020; Saito et al., 2021), drugs (Barre et al., 2016), amines (Chacon et al., 2011; Manier et al., 2014), fatty acids (Wu et al., 2016; Wang et al., 2019; Iwama et al., 2021), amino acids (Toue et al., 2014; Esteve et al., 2016; Guo et al., 2020), poisons (Beasley et al., 2016), plant hormones (Enomoto et al., 2018), peptides (Franck et al., 2010), steroids (Guo et al., 2020; Angelini et al., 2021; Song et al., 2021), neurotransmitters (Ito and Hiramoto, 2019; Palanisamy et al., 2020; Shariatgorji et al., 2020), and so on. It is worth noting that the coating method of derivatization solution and matrix is also important because it affects not only the efficiency of OTCD but also the quality of imaging results.

If the derivatization solution also serves as a matrix (reactive matrix), then no additional step is required. Reactive matrix can also reduce the double interference of excessive chemical derivatization solution while reducing matrix effect. Recently, Shariatgorji et al. designed a reaction matrix based on fluoromethylpyridine that can promote covalent charge labeling of molecules containing phenolic hydroxyl groups and/or primary or secondary amine groups, including dopaminergic and serotonergic neurotransmitters (Shariatgorji et al., 2019). The matrix improves the detection limit of MALDI-MSI for low-abundance neurotransmitters and realizes the simultaneous imaging of neurotransmitters in the fine structure of the brain (Shariatgorji et al., 2019). Davison et al. used 2,4-diphenyl-pyran tetrafluoroborate (DPP-TFB) to react with monoamine neurotransmitters and directly measured the content in the brain tissue of mice by MSI (Davison et al., 2019). In addition, 2,4,6-trimethylpyridine tetrafluoroborate (TMP-TFB)-derived matrix MALDI-MS can image dopamine in mouse brains (Wang et al., 2021). Girard’s T reagent and TAHS, respectively, enhanced the ionization efficiency of steroids and catecholamine (Takeo et al., 2019).

#### 3.3.3 Organic Matrix-Free Inorganic Nanomaterials as Matrices

**FIGURE 4:**
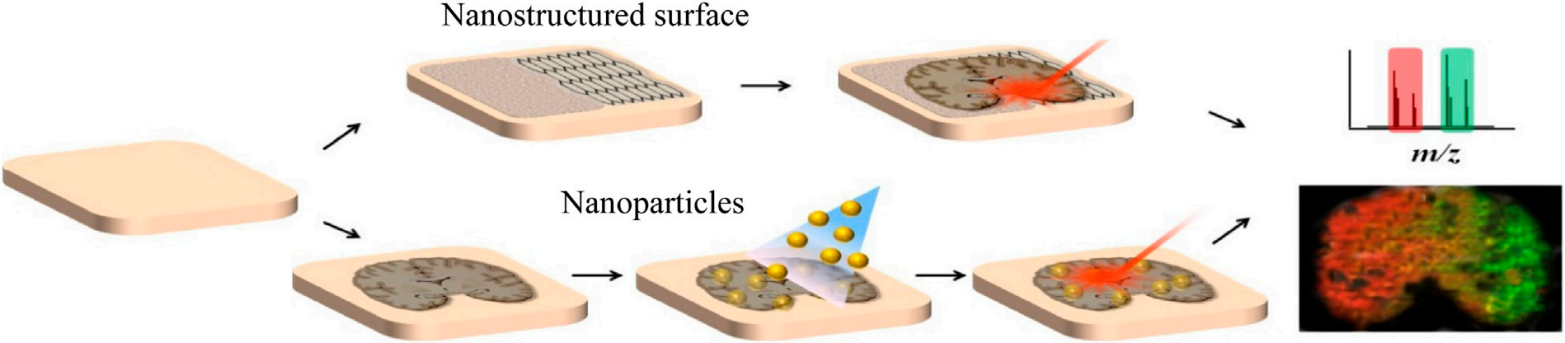
LDI MSI with organic matrix-free systems harnessing nanostructured surfaces or nanoparticles (Kim et al., 2020).

Inorganic materials can also be used in the application of small molecules in MALDI because they are not easily ionized, which will not interfere with analytes as traditional matrices. Recently, there is an increasing trend to use nano-structured surfaces and inorganic nanoparticles as substitutes for organic matrices and develop various organic-free MSI systems (Figure 4). For example, Rudd et al. used the desorption–ionization of porous silicon (DIOS) nanomaterials to study the changes in biodistribution during the reproductive cycle and found that muscle relaxation choline ester murexine and tyrosine sulfate colocalize in the lower branchial glands (Rudd et al., 2015). Carbon-based surfaces can also be used for matrix-free MSI. Kim et al. used graphene oxide (GO)/multiwalled carbon nanotube (MWCNT)-based films as a new matrix-free laser desorption/ionization platform with efficient analyte desorption/ionization, minimal fragmentation, high salt tolerance, excellent durability, and other advantages, suitable for tissue imaging mass spectrometry (Kim et al., 2011). Bien et al. cultured the cell line directly on indium tin oxide (ITO)-coated glass slides and used transmission (t-) mode MALDI-2-MSI to analyze the 2-μm pixel size culture, which can visualize the spatial distribution of dozens to hundreds of different biomolecules in tissue section and cell culture (Bien et al., 2021a). AuNPs and AgNPs are the most commonly studied and widely used nanoparticles in MSI, and they have the characteristics of easy adjustment of dimensional properties. McLaughlin et al. developed a neurotransmitter ionization method based on AuNPs, which sprayed AuNPs on tissue slices by air pressure and can perform mass spectrometry imaging of a variety of tissues and realized the localization of neurotransmitters in zebrafish embryos and neuroblastoma cells, with a horizontal spatial resolution of 5 μm (McLaughlin et al., 2020). Han et al. synthesized polydopamine (PDA)-encapsulated AgNPs (AgNPs@PDA) as the matrix of MALDI MSI to analyze lipids in positive and negative ion mode, and controlled the signal of silver cluster ions by adjusting the thickness of the PDA layer (Han et al., 2019). It was found that with AgNPs@PDA as the matrix, the PC signal was greatly inhibited, while other lipids (including PE, HexCer, PS, PI, PIP, and ST) were, on the contrary, achieving the detection of 58 glycerophospholipids and 25 sphingomyelins in brain tissue slices.

#### 3.3.4 Room-Temperature Ionic Liquid Matrix

During recent years, one kind of new green solvents, room-temperature ionic liquids (ILs), that remain liquid at room temperature have received much attention to replace current harsh organic solvents due to their distinct properties and characteristics compared with traditional solvents. Usually, ILs are composed of relatively bulky organic cations and relatively small inorganic anions and have a melting point below 100°C, and they are stable at temperatures below 250°C (Tholey and Heinzle, 2006). In addition, ILs have inherent properties, such as negligible vapor pressures, low volatility at room temperature, and high thermal stability, which, in turn, contribute to their recovery and reusability in separation and purification processes. So now ionic liquids have been widely applied in different research and industrial fields, such as chemistry, biology, catalysis, energy, and even environmental sciences (Hallett and Welton, 2011; Itoh, 2017; Ventura et al., 2017; Huang et al., 2019).

Due to their low vapor pressure, the ability to dissolve a wide range of substances, ionic liquids have been applied as matrix in MALDI, called ionic liquid matrix (ILM). ILM is synthesized from conventional MALDI matrix compounds (such as DHB, CCA, and SA) and organic bases [such as pyridine (Py), tributylamine (TBA) and N, N-dimethylethylenediamine (DMED)]. One of the most striking advantages for applying ionic matrix in MALDI-MSI is that sample homogeneity can be achieved because the viscous liquid surface is highly homogeneous. Compared with solid matrices, ILM has good vacuum stability, good reproducibility, and high sensitivity (Armstrong et al., 2001). For example, the analysis of lipid can be enhanced by applying ILM in MALDI. Wang et al. used different ratios of 9-aminoacridine (9-AA) and N-(1-naphthyl) ethylenediamine dihydrochloride (NEDC), two matrices with orthogonal selectivity for the ionization of lipids (Wang et al., 2018). After mixing, the mouse brain lipid extract was analyzed, and the final detection range was enhanced.

#### 3.3.5 New Spraying Method to Add Organic Matrix

Matrix application needs to be uniform, produce small crystal sizes, and appropriately extract analytes without introducing artifacts such as spatial delocalization. It is economical and easy to operate using a sieve and manual spraying with airbrushes (Yang et al., 2012). The first sublimation device for matrix deposition reported by Hankin et al. can produce a uniform layer of small crystals on the sample plate (Hankin et al., 2007). This method is easy to control and has high repeatability, and can obtain high-quality mass spectrometry image. In addition, compared with the sublimation recrystallization method and sublimation only, the lipid ion signal intensity of the pneumatic matrix spraying method samples increased by 8 and 30 times on average in the experiment of Kompauer et al. (2017). Therefore, it is suggested to use pneumatic sprayers or sublimation devices to reduce errors.

### 3.4 Data Acquisition, Processing, and Visualization

After MALDI-MS analysis, MSI software is needed to control data acquisition, processing, and integration in order to generate ion imaging. There are many commercial and open-source software that can be used to process MSI data, such as Biomap (Stoeckli et al., 2002), FlexImaging, MALDI Imaging Team Imaging Computing System (MITICS), Datacube Explorer, ClinPro Tools (Ketterlinus et al., 2005), and so on. A comparison of several developed software for MSI was presented by Kamila Chughtai and Ron M.A. Heeren (Chughtai and Heeren, 2010). As the MSI data contains massive information including mass spectrometry information and spatial information, the resolution and spatial resolution have also been continuously improved with the development of MSI, which leads to large amounts of original imaging data, and it became increasingly difficult to process it.

The quality of MSI images can be improved by removing noise, correcting deviation of *m/z* peak, and normalization. The purpose of normalization is to reduce the signal difference between pixels that may be caused by disconformity matrix coating and ion suppression. Normalization based on total ion count (TIC) and vector norm normalization are currently the most commonly used methods. However, regarding potential biomarker distributions, other normalization algorithms may be needed to prevent producing misleading results (Deininger et al., 2011).

Data analysis considerations for 3D-MSI data analysis is very important after data normalization (Vos et al., 2021). Benchmark datasets for 3D MALDI-MSI provide high-quality 3D imaging datasets from different biological systems at several labs, which stimulates computational research in the field of computational 3D imaging MS (Oetjen et al., 2015). Nowadays, principal component analysis (PCA), hierarchical cluster analysis (HCA), and partial least square discriminate analysis (PLS-DA) are the most common multivariate statistical analysis methods used in MSI. These methods are successful in dimension reduction and feature extraction. The factor analysis method was studied, and it was proved to be able to simply and quantitatively extract the target sample markers (Chen et al., 2014).

### 3.5 Quantitative on-Tissue Matrix-Assisted Laser Desorption/Ionization-Mass Spectrometry Imaging Process

**FIGURE 5:**
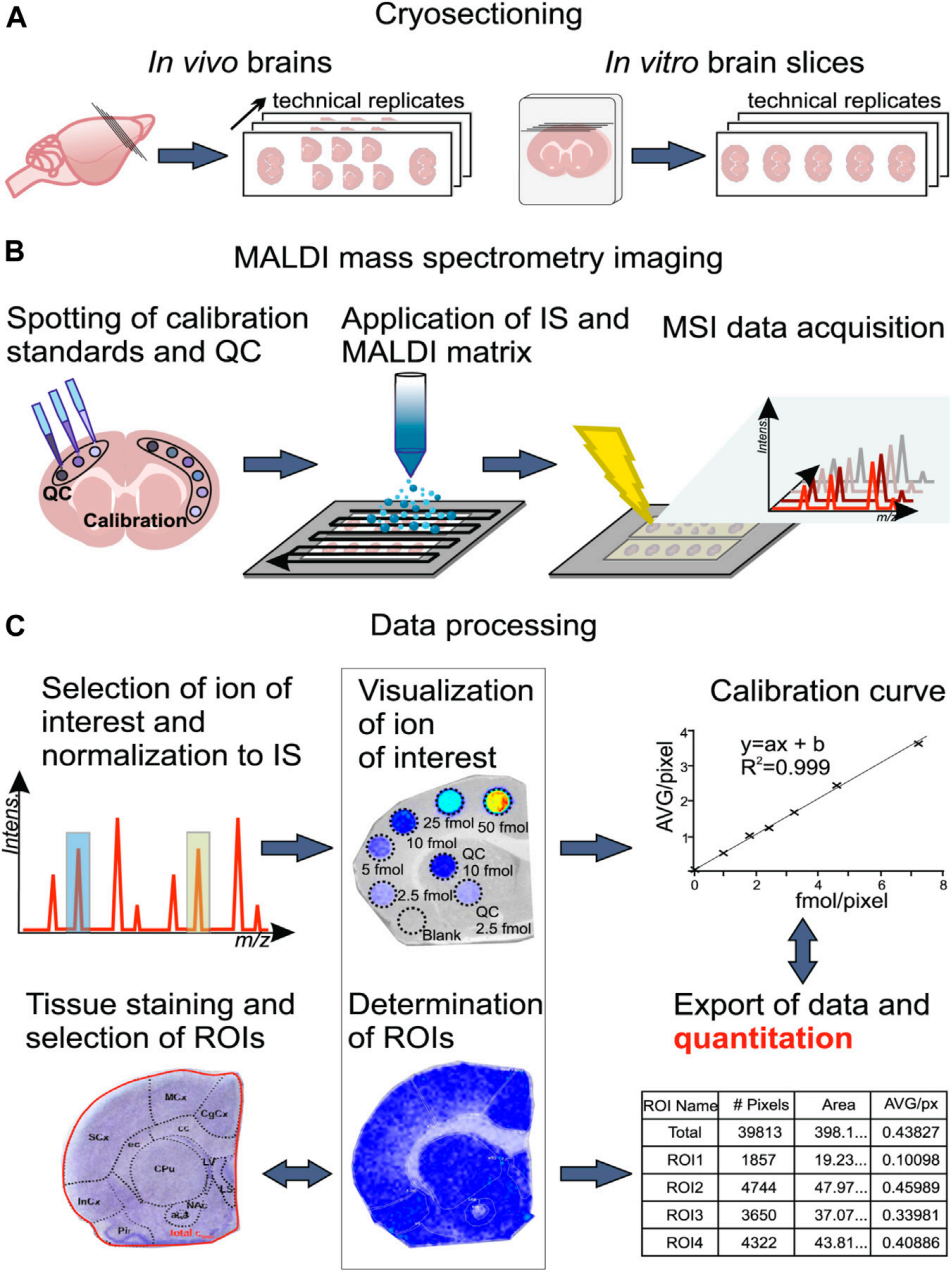
Illustration of the workflow for assessing total brain drug concentrations using qMSI-uD (Luptakova et al., 2021).

On-tissue quantitative determination of biomolecules or administrated drugs is very important for MSI analyses. For example, it is essential to determine the extent of drug transport across the region-specific blood–brain barrier (BBB) and discriminate the regional free (unbound) drug concentration at which the drug engages with its therapeutic target. Recently, a new method, qMSI for unbound drug determination (qMSI-uD), combining *in vivo* and *in vitro* neuropharmacokinetic studies with MSI, has been developed to assess the extent of unbound drug transport across the BBB and drug distribution in small anatomical regions (including subregions) in the brain (Figure 5). Using this method, direct imaging of three antipsychotic drugs (risperidone, clozapine, and olanzapine) with different BBB transport properties and regional distribution patterns was performed at 20-µm resolution. In addition, the method provides region-specific drug exposure data associated with drug response data, facilitating development of new drugs (Luptakova et al., 2021).

## 4 Single-Cell Imaging

Cells in multicellular organisms have different morphological and gene expression patterns. Cell phenotypic transition occurs during the development of the fertilized egg into different cell types, as well as under the physiological and pathological conditions of the differentiated cell. Heterogeneity among cells underlies individual variability in the activity of cellular networks and circuits (Goaillard et al., 2009). Due to the morphological, physiological, and pathological heterogeneity of cells, there is currently much interest in broad molecular profiling of a single cell. It is very important to study the biochemical and physiological characteristics of individual cells and their environment. Analysis of single cells can further our understanding of differential susceptibility to treatment of disease (Rubakhin et al., 2011). However, previous techniques have difficulties in capturing the information of cell phenotypic transition dynamics. Fortunately, live-cell imaging and analysis fill this gap (Wang et al., 2020). MSI can reveal the distribution of hundreds of compounds simultaneously in the cell and tissue sections down to single-cell level (Duenas and Lee, 2021).

It should be pointed that the size of the cell is small, and thus, a higher resolution is required. For human cells, the diameter of a cell ranges from 5 μm (sperm cell) to 150 μm (ovum) (Gillooly et al., 2015; Ginzberg et al., 2015) and, on average, about 10–20 μm. In some diseases, the average size of the affected cells will change, such as cancer cells, which are commonly larger than their respective normal cells, and usually, cells of different sizes are mixed. For example, cancer cells are often surrounded by smaller infiltrating lymphocytes (Stern et al., 2017). So, the needed minimum spatial resolution is determined by the smallest cell size or distance between cells (Scupakova et al., 2020). In initial MALDI single-cell imaging experiments, large frozen *A. californica* cells could be imaged at a 50-μm raster size, but for much smaller mammalian cells, MALDI-MSI needed to pursue higher resolutions (Lanni et al., 2012).

### 4.1 Single-Cell Metabolomics

Single-cell metabolomics provides insight into phenotypic variation between individual cells. Changes in metabolite concentrations and differences in lipid and protein profiles lead to unique metabolome profiles for individual cells (Scupakova et al., 2020). Metabolome molecular profiles can provide the most accurate information of cellular reaction networks, helping to understand the link between genotypes and phenotypes of individual cells. Existing microfluidics, micromanipulation, image analysis, and automation technologies have enabled high-throughput isolation of individual cells with minimal interference without affecting cell metabolism (Ali et al., 2019).

Up to now, single-cell MSI technology has been successfully used to obtain data from individual plant cells, which helps to reveal unprecedented insights on metabolic outcomes. It was commonly used to investigate the differential location and heterogeneity of secondary metabolites in plant tissues. For example, MALDI-MSI of maize leaves clearly indicated the distribution of two major anionic lipids in thylakoid membranes, which helps to reveal the genetically programmed and developmental modification of thylakoid membrane (Duenas et al., 2017b). High-resolution MS of individual lipid droplets from cotton seed tissues helps to understand the cellular context of lipid origin (Horn et al., 2012).

In addition, single-cell imaging can help to identify the colocalization of the distribution of individual molecular species, including particular lipids and proteins, and correlation with the morphological features of a tissue section, which also plays an important part in molecular pathology and cancer therapy. For instance, a single-cell MALDI-MSI approach revealed a decreased level of phosphatidylcholine (16:0/20:4) in multiple myeloma cells compared with plasma cells (Hossen et al., 2015). MSI is also a useful tool for testing chemotherapeutic drugs and drug combinations in cancer therapy, making it possible to monitor drug response in primary cancer spheroids (Mittal et al., 2019). The sensing ability of this method can be improved in single cancer cells and cancer stem cell analysis through nano-platform-mediated microwave digestion (Manikandan and Wu, 2016).

Recently, an open-source single-cell metabolomics method named SpaceM, which integrates light microscopy and MALDI-MSI has been developed for in situ detection of >100 metabolites from >1,000 individual cells per hour together with a fluorescence-based read out and retention of morphological spatial features based on fluorescence (Rappez et al., 2021). In addition, SpaceM can easily distinguish between cells in a state. SpaceM The SpaceM method includes following four steps. Firstly, cell segmentation of the microscopy images provide a broad panel of phenotypic information including fluorescence intensities and morphological individual cells. Next, MALDI-MSI is performed for untargeted detection of metabolisms. Then, MALDI pixel registration and single-cell intensity normalization are performed, which compensate for differences in cell sampling and filter out ambiguous ablation marks sampling multiple cells. Finally, SpaceM provides a matrix with a multiplex readout which comprises an untargeted metabolic profile, fluorescence intensities and spatio-morphological features, thus it integrates metabolism profile and phenotype.

### 4.2 Single-Cell Molecular Mapping

The regionalization of biological functions is a fundamental phenomenon of life, and this regionalization can be observed at different levels, ranging from organs to specific cells and even subcellular structures. At single-cell level, proteins and metabolites play a role in a specific time and space, which provides a specific chemical environment and interaction factor. Thus, understanding the subcellular localization is necessary to further study the biological process. However, there are several factors that hinder the development of single cell molecular mapping at subcellular level, such as the analyte delocalization caused by complex sample preparation process and, thus, hampering high spatial resolution, loss of molecular information due to the increase in spatial resolution, and the difficulties in handling processing, integration, and storage because of the increase in data size (Scupakova et al., 2020).

Recent advances in sample preparation, instrumentation and data processing have led the MALDI-MSI molecular mapping to approach subcellular level. An atmospheric pressure (AP) MALDI-MSI setup might achieve imaging of tissues and cells at a lateral resolution of 1.4 µm, a mass resolution greater than 100,000, and accuracy below 62 ppm (Kompauer et al., 2017).

**FIGURE 6:**
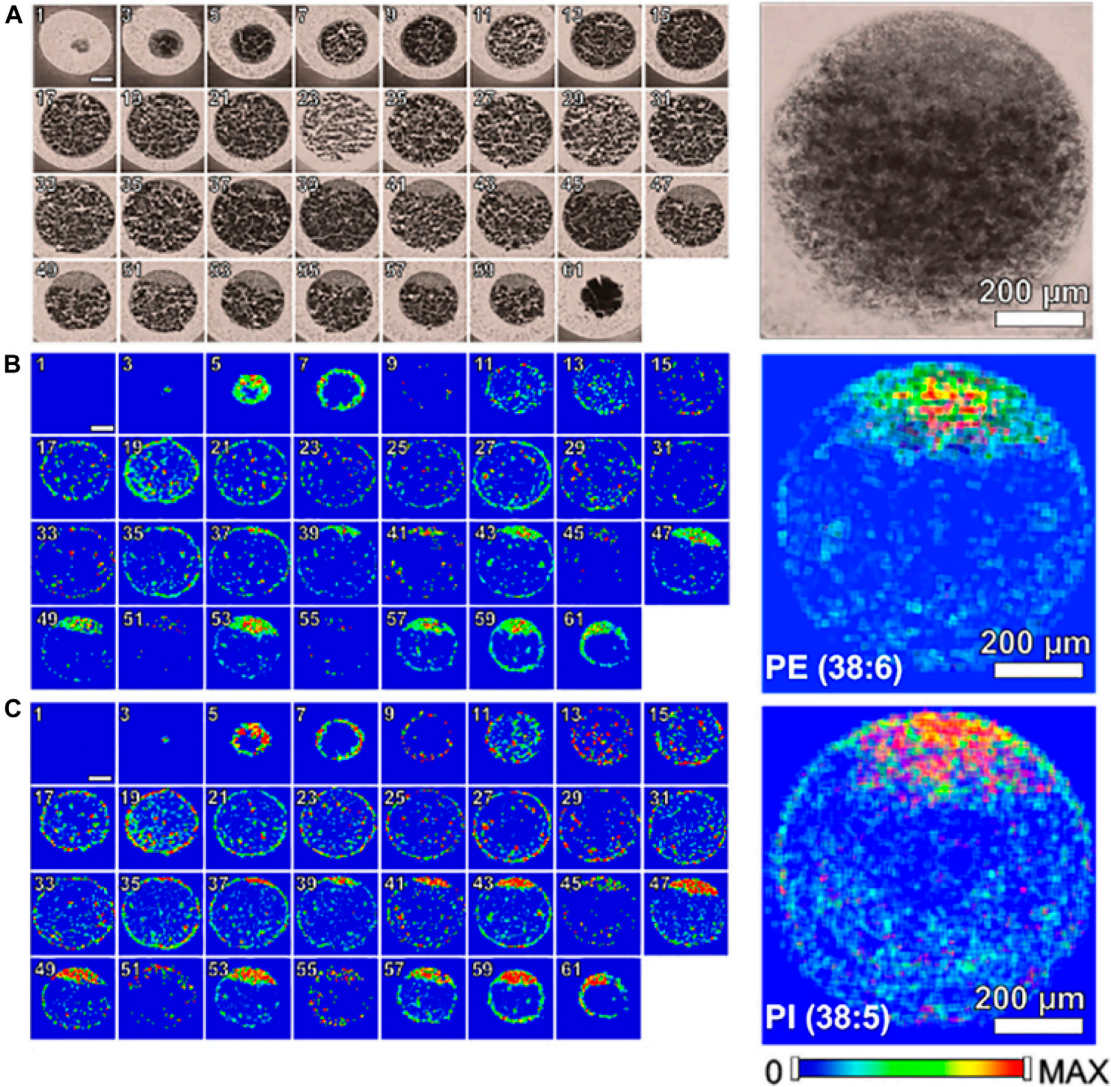
Single cell molecular mapping Zenobi, 2013. Odd numbered optical images of fertilized zebrafish embryo at the one-cell stage, false color two-dimensional MALDI-MS images of PE (22:6_16:0) at *m/z* 762.509 and PI (18:0_20:5) at *m/z* 883.535, and projected images are shown on the right by overlaying all 2D images (Duenas et al., 2017a).

Zebrafish, a small tropical aquarium fish native to Southeast Asia, have a unique combination of genetic and experimental embryologic advantages that make them as an ideal model vertebrate organism for studying and understanding developmental biology, drug discovery, and neurodegenerative diseases especially for early development. Recently, a study (Duenas et al., 2017a) applied high-spatial resolution MALDI-MSI to map and visualize the 3D chemical imaging of a single cell for spatial distribution of phospholipid classes, phosphatidylcholine (PC), phosphatidylethanolamines (PE), and so on, in newly fertilized individual zebrafish embryos. As shown in Figure 6, MALDI-MS images of PE (22:6_16:0) and PI (18:0_20:5) show that PE and PI are mostly absent or present minimally inside the yolk. In addition, to better understand how the metabolites may change when the zebrafish embryo develops, a number of embryos at different stages (1-, 2-, 4-, 8-, and 16-cell stage) were evaluated using high-spatial resolution 2D MALDI-MSI (Duenas et al., 2017a).

### 5 Two-Dimensional on-Tissue Mapping Molecular Distribution

Histopathological examination of tissues and cells provides clinically important and necessary insights. Visual inspection, which relies on stained tissue sections, is a classic pathological examination. The ability of MALDI-MSI to provide spatial location information is one of its great advantages. Now combining the analytical capabilities of MS with the benefits of microscopy to analyze molecular events occurring in specific cell types in tissues will take anatomic pathology a big step forward (Norris and Caprioli, 2013b).

### 5.1 Pathological Classification

Pathological classification of tumor cells is a difficult problem due to the similarities of different tumor (sub) types. Because assessment is usually performed manually, the results may be subject to human error. MALDI-MSI can determine the spatial distribution of multiple compounds (lipids, peptides, and proteins) in complex tissues in a single, label-free measurement. Especially in cancer research, spatial protein characterization of tissue and biomarker identification will lead to better diagnosis and individual predictive patterns of therapy response. Now, MALDI-MSI combined with machine learning has been used to classify various cancers including renal oncocytoma, clear cell renal cell carcinoma, and chromophobe renal cell carcinoma, and results showed that MSI correctly classified 87% of patients (Möginger et al., 2020). In addition, the major advantages of the method classifying cancer subtypes also simultaneously reveal the molecular features of cancer cells.

**FIGURE 7:**
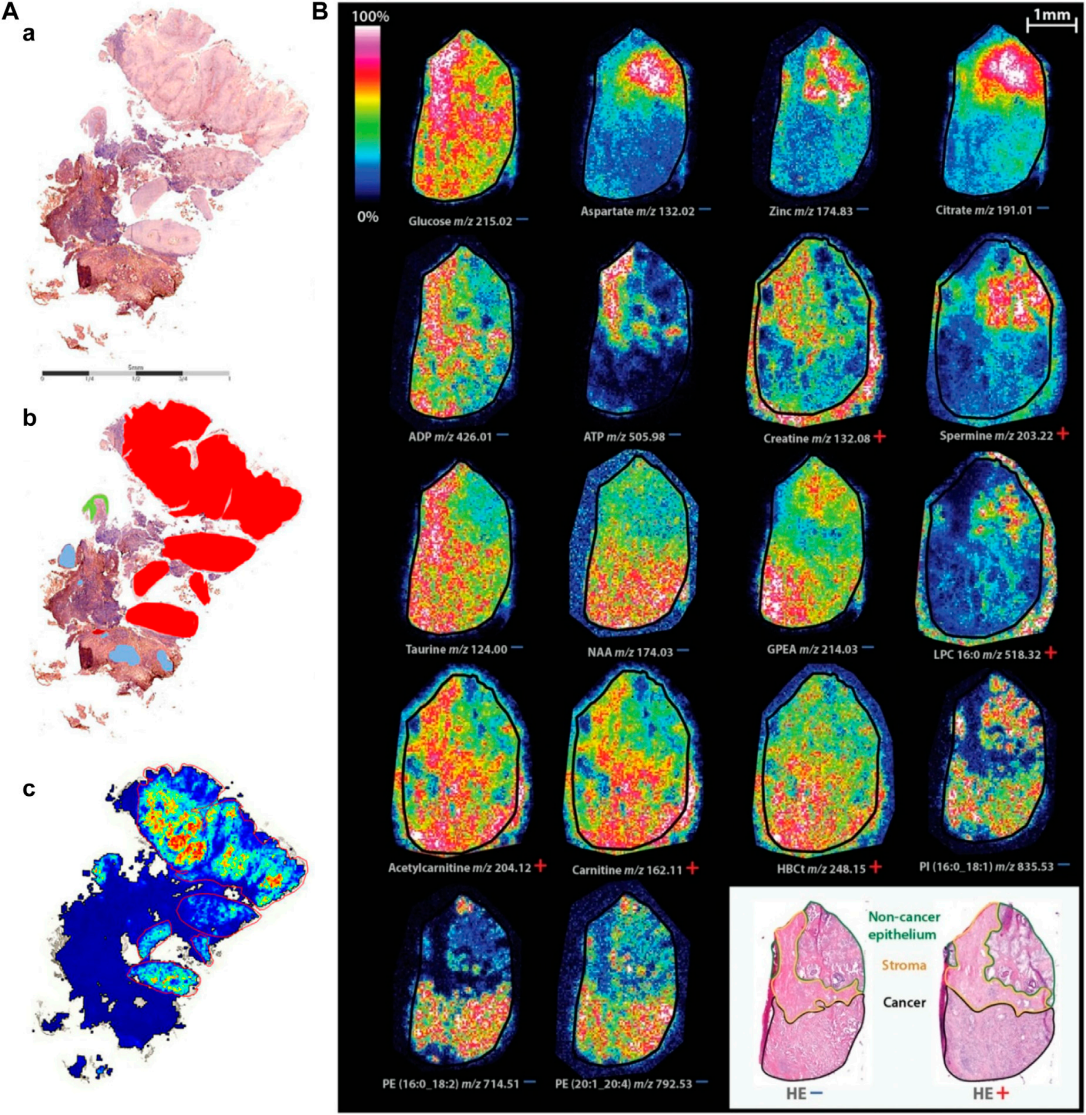
Typical characterization of cancer and biomarker by MALDI-MSI. **(A)** Comparison of component analysis of MALDI-MSI measurements on FFPE sections with histopathological regions. One example of sample cohort is shown. (a) Hematoxylin and eosin-stained tissue section. (b) Annotated regions, red: squamous cell carcinoma, green: dysplastic epithelium, rest: nontumor region, containing connective tissue with inflammatory infiltration and glandular regions (blue). (c) Component that covers the tumor region; no spatial denoising was performed (Hoffmann et al., 2019). **(B)** Spatial distribution of identified masses in both ion modes on consecutive tissue sections (Andersen et al., 2021).

As shown in Figure 7A, although the heterogeneity of tumor tissue complicates diagnosis and individualized treatment, MALDI-MSI still can clearly discriminate tumor regions from nontumor regions by simultaneous detection and location of multiple protein markers (Hoffmann et al., 2019). A recent study indicated that MALDI-MSI could be used to directly detect excessive hormonal production from functional pituitary adenomas and generally classify pituitary adenomas by using statistical and machine learning analyses. The tissue characterization can be completed in fewer than 30 min and could, therefore, be applied for the near real-time detection and delineation of pituitary tumors for intraoperative surgical decision making (Calligaris et al., 2015). Therefore, clinical MALDI-MSI is helpful for the analysis of tumor tissue during surgery and can provide precise digitized diagnosis for intraoperative decision making.

### 5.2 Biomarker Discovery and Distribution

In general, for tumor diagnosis, biomarkers are used to distinguish between tumor cells and normal cells. MALDI-MSI can assist in the identification of lipid profile differences in breast cancer tissues, and it was found that phosphatidylcholine and triacylglycerol were the main compounds detected in cancer and normal areas as biomarkers. Very high intensity of the triacylglycerol ion signals were detected in the normal tissue region, whereas very strong ion signals of phosphatidylcholine were detected in the tumor tissue region (Cho et al., 2017). MALDI-MSI of normal and tumor areas can clearly see different lipid patterns, improving the accuracy of breast cancer diagnosis. Similar results were found in MALDI-TOF-MSI analysis of the lipid profile of prostate cancer, which showed that prostate cancer was related to the synthesis of fatty acids and lipid oxidation, while PC 16:0/16:1, PC 16:0/18:2, PC 18:0/22:5, PC 18:1/18:2, PC 18:1/20:0, PC 18:1/20:4, and SM d18:1/24:0 can be used as good biomarkers (Buszewska-Forajta et al., 2021). In addition, a form of medullary thyroid carcinoma (MTC) progresses from C-cell hyperplasia (CCH). The proteome changes in MTC and CCH tissues were analyzed by MALDI-MSI. The results showed that the trypsin profiles of MTC and CCH were significantly different, and there were four MTC markers available, K1C18 and three histones (H2A, H3C, and H4). Therefore, MALDI-MSI is a new proteomic tool that can be used to identify new molecular markers for diagnostic and prognostic significance (Smith et al., 2019).

As shown in Figure 7B, using MALDI-TOF-MSI, significant metabolic changes were found in relation to lipid metabolism and prostate secretory function between noncancer epithelium, stroma, and tumor. Elevated levels of metabolites associated with lipid metabolism in tumor include carnitine shuttle, which facilitates fatty acid oxidation, and metabolites of building blocks required for lipid synthesis. Levels of metabolites associated with prostate function, including citrate, aspartic acid, zinc, and spermine were higher in noncancer epithelium than in tumor. The stroma had higher levels of important energy metabolites (such as ADP, ATP, and glucose) and higher levels of the antioxidant taurine than the other. This study showed that specific tissue compartments of tumor have different metabolic profiles. Spatial metabolic profiling helps in precision therapy and potential biomarker discovery (Andersen et al., 2021).

Besides small-molecule biomarkers, such as lipids and phosphatidylcholine, there are some peptides and proteins to be found as diseases biomarkers. For example, a recent study (Balestrieri et al., 2021) indicated the signal intensity of galectin1 peptides in lung metastases compared with adjacent normal tissues and control lung. Moreover, the most intense peptide signals were found at the edges of metastases compared with adjacent normal lung tissues. MALDI-MSI can be applied to the application of proteomics methods in cancer research, especially in the spatial distribution of tumor cells.

Understanding the causes will facilitate targeted treatment and more appropriate allocation of medical resources. As for chronic kidney disease, there are two most common causes, diabetic nephropathy and hypertensive nephrosclerosis. Using MALDI-FTICR MS and nLC-ESI-MS/MS, it is speculated that four detected proteins with high signal intensity in the diabetic nephropathy tissue (PGRMC1, ANXA5, CO3, and LDHB) could be used as biomarkers to reliably distinguish the cause of CKD. Moreover, the signal intensity of PGRMC1 and CO3 increased in the late stage of the disease, which may be related to the progression (Smith et al., 2020).

### 5.3 Mapping Molecular Distribution

#### 5.3.1 Imaging the Neurotransmitters

Acetylcholine (ACh) is an important neurotransmitter involved in neurodegenerative disorders. A study revealed age-related changes in acetylcholine levels in normal mice treated with the acetylcholinesterase inhibitor drug tacrine. Using MALDI-MSI, tacrine was found to significantly increase acetylcholine levels in most brain regions of mice. However, after administration, acetylcholine levels in retrosplenial cortex of 14-month-old animals were significantly lower than those of 12-week-old animals, suggesting that normal aging affects the reactivity of the cholinergic system. The distribution of tacrine and its hydroxylated metabolites in the brain was also observed, and the metabolite levels decreased significantly in 14-month-old mice. The results highlight the advantages of imaging techniques that can simultaneously investigate multiple molecular species and specific regions of drug target effects (Vallianatou et al., 2019).

**FIGURE 8:**
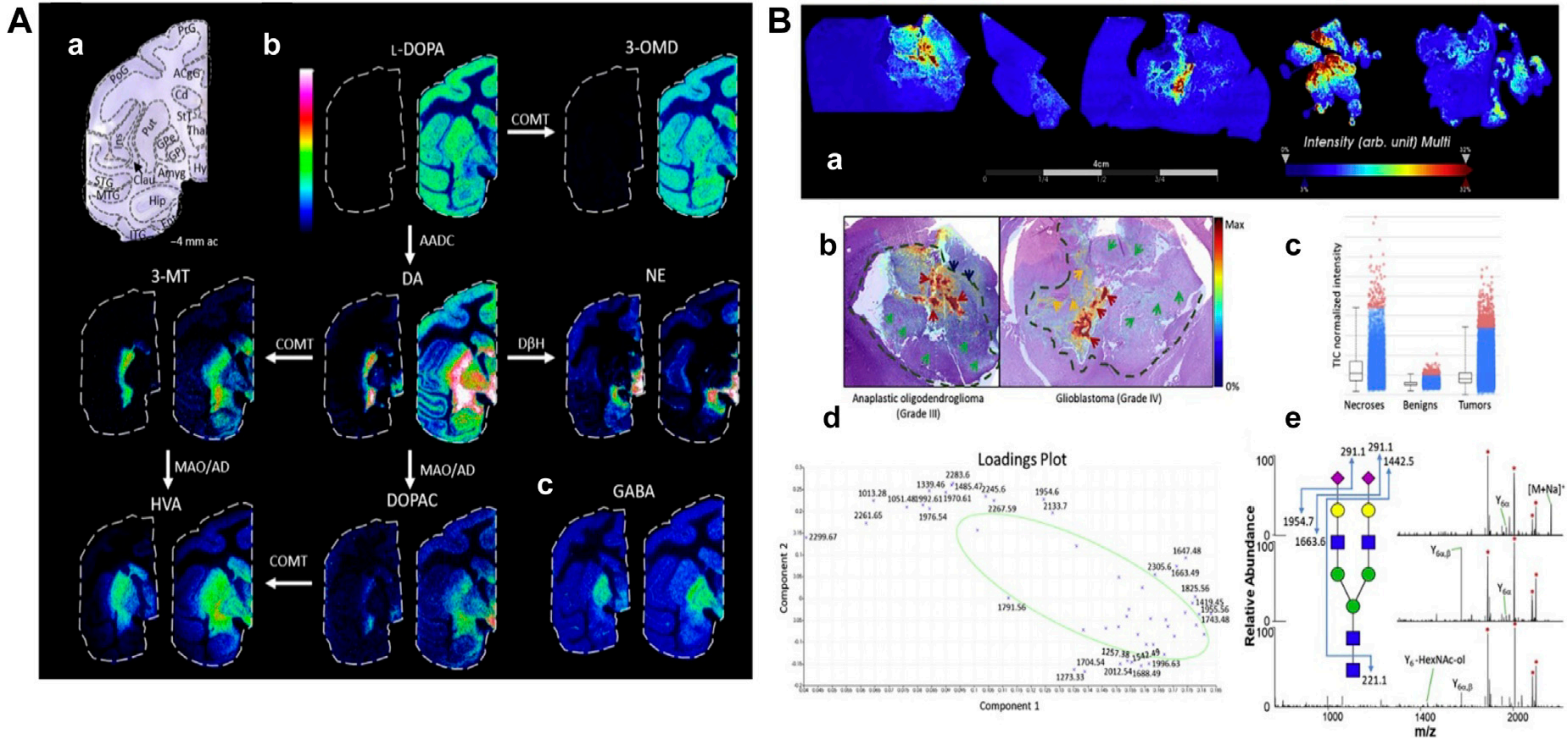
Mapping typical molecular distributions of **(A)** neutrotransmitters, **(B)** N-glycoproteomes. **(A)** MALDI-MS images of neurotransmitters and metabolites in non-LID and LID. (a) Nissl-stained macaque brain tissue section at −4 mm ac with annotated brain regions. (b) Catecholaminergic metabolic pathway. (c) GABA (Fridjonsdottir et al., 2021). **(B)** Spatially resolved N-glycans by MALDI-MSI. (a) Summed ion images of Na^+^ and K^+^ adducts of HexNAc_4_-Hex_5_-NeuAc_2_ on canine glioma biopsies. (b) Superposition of MALDI-MSI glycan images with H&E-stained adjacent sections. Arrows and dashed lines indicate regions annotated by the pathologist. (c–e) Normalized intensity of total ion signals, PCA analysis, and MS^n^ spectra of HexNAc4-Hex5-NeuAc2 (Malaker et al., 2021).

L-DOPA therapy for Parkinson’s disease often leads to dyskinesia. The distribution of L-DOPA and monoaminergic pathways in the brains (Figure 8A) of dyskinetic and nondyskinetic primates was mapped using MALDI-MSI. The levels of L-DOPA and its metabolite 3-O-methyldopa were increased in all measured brain regions of dyskinetic animals, and the levels of dopamine and metabolites were increased in all analyzed regions except the striatum. The level of dopamine was significantly correlated with the level of L-DOPA in extrastriatal regions. L-DOPA-induced dyskinesia is associated with whole-brain L-DOPA dysregulation. High dopamine abundance in extracranial regions may alter signal transduction throughout the brain, leading to various adverse effects of L-DOPA treatment (Fridjonsdottir et al., 2021).

#### 5.3.2 Imaging N-Glycoproteomes

Aberrant glycosylation is a common feature of cancer. MALDI-MSI has been used to study changes in N-glycosylation in cancer, using a combination of MALDI N-glycan MSI and spatially resolved glycoproteomics. Thus, glycosylation imaging (Figure 8B) is directly linked to complete glycopeptide identification. This glycoproteomics technique identified more than 400 N-, O-, and S- glycopeptides from more than 30 proteins. The sialylated O-GalNAc structure was significantly increased in the tumor/necrotic area compared with the benign area, while S- and O-GlcNAc peptides were significantly decreased in the cancerous area. This experiment provides a unique way to understand the spatial variability of glycosylation changes in cancer (Malaker et al., 2021).

#### 5.3.3 Imaging Host–Microbe Symbioses

Symbioses are widespread in nature. There are complex biochemical interactions between them which affect each other, and MALDI-MSI can help us further study about the relationship between host and microbe.

**FIGURE 9:**
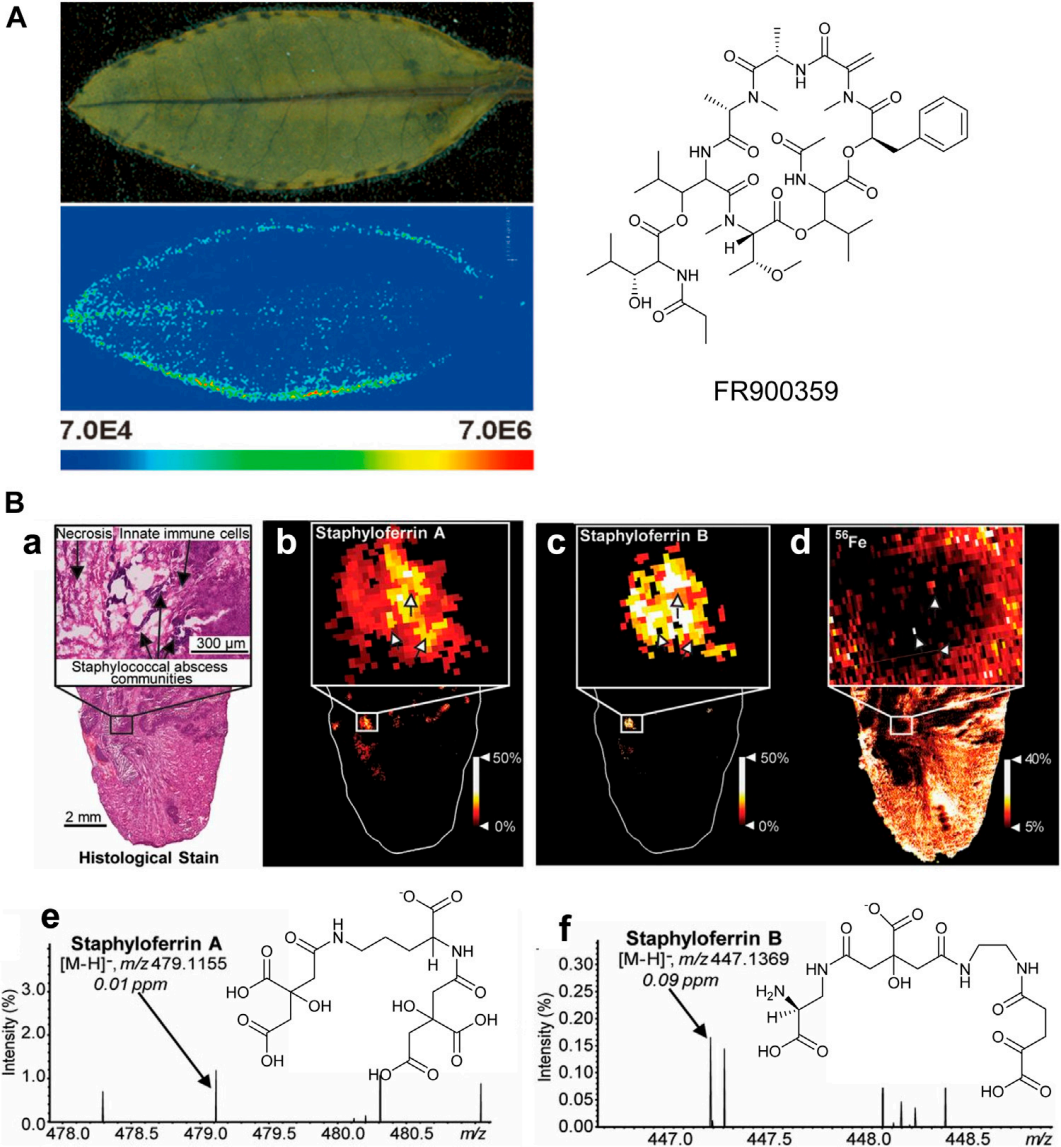
**(A)** MALDI imaging mass spectrometry of an Ardisia crenata leaf (Cruesemann et al., 2018). **(B)** MALDI-IMS reveals siderophores staphyloferrin A (SA) and staphyloferrin B (SB) within the infectious environment. (a–d) The distributions of SA and SB with SACs. (e-f) The signals and the chemical structures of SA, [M-H]^−^ and SB, [M-H]^−^ (Perry et al., 2019).

When a biological symbiosis is mutually beneficial, it is termed “mutualism.” For example, obligatory plant–bacteria associations, as observed in the case of the nodulated *Ardisia crenata*, constitute fascinating ecological systems (Carlier et al., 2016). The cyclic depsipeptide FR900359, a strong and selective inhibitor of Gq proteins, is isolated from the tropical plant *Ardisia crenata* (Fujioka et al., 1988), but it is finally found to be produced from the symbiotic “Candidatus *Burkholderia crenata*,” a bacterium that is mostly located in the nodules at the leaf margin of *A. crenata* (Carlier et al., 2016), which is consistent with recent MALDI-MSI results (Figure 9A). The small blue dots show the distribution of FR900359 (*m/z* 1,040.49), which corresponds to “Candidatus *Burkholderia crenata*” at the margin of *A. crenata* leaves, suggesting that FR900359 plays a novel mode of action for defense chemicals through Gq inhibition (Cruesemann et al., 2018).

There is a parasitic relationship between *Staphylococcus aureus* and vertebrate hosts. *Staphylococcus aureus* feeds on the nutrients of the host, which is another type of symbiotic relationship. Typically, the metalloproteins in the host isolate the very important metal elements in the body to prevent the absorption of microorganisms during infection. However, bacteria have also evolved metal acquisition strategies to combat nutritional immunity, such as the use of siderophores and small iron-scavenge molecules. Recent studies have used multimodal MALDI-MSI to image siderophores in infected tissues to visualize host–pathogen iron competition (Perry et al., 2019). It can be observed that the heterogeneous distribution of *Staphylococcus aureus* siderophores across the infected lesions is observed, as shown in Figure 9B. These results suggest that each siderophore has a niche-specific role, rather than functional redundancy. Differential distributions of these siderophores may be explained by molecular heterogeneity within the abscess.

More recently, a spatial metabolomics pipeline (metaFISH) has been developed by combines fluorescence in situ hybridization (FISH) microscopy and high-resolution atmospheric-pressure MALDI-MSI in order to image host-microbe symbioses and its metabolic interactions and provide spatial assignment of host and symbiont metabolites. The metaFISH workflow consists of three steps. Firstly, mapping metabolites with high-resolution AP MALDI-MSI on cryosections and FISH after MSI on the same tissue section. Then, spectral preprocessing, image adjustment, cluster maps, phylotype assignment is needed to process correlative data. Finally, statistical analysis is performed using the fluorescence signals to bin metabolite groups. This method presented the spatial metabolome of a deep-sea mussel and its intracellular symbiotic bacteria, revealing the metabolic adaptability of epithelial cells to intracellular symbionts and metabolic phenotypic variation of the 16S rRNA phylotype of an individual symbiont, and making it possible to discover specialized metabolites from the host–microbe interface (Geier et al., 2020).

## 6 Three-Dimensional Spatial Imaging

Since biological processes take place in three-dimensional organisms, it is not surprising that 3D imaging has a noteworthy impact on different studies in life sciences. Recently, the use of MSI to image intact biomolecules has been extended to 3D analysis to determine the volumetric molecular distribution in tissue samples. The most common 3D MSI method includes collecting consecutive tissue sections of the samples, analyzing each section separately using traditional two-dimensional MSI, and then using computational methods to stack and reconstruct the final 3D MSI dataset from multiple two-dimensional MSI data.

### 6.1 Three-Dimensional Reconstruction of Spatial Distribution

**FIGURE 10:**
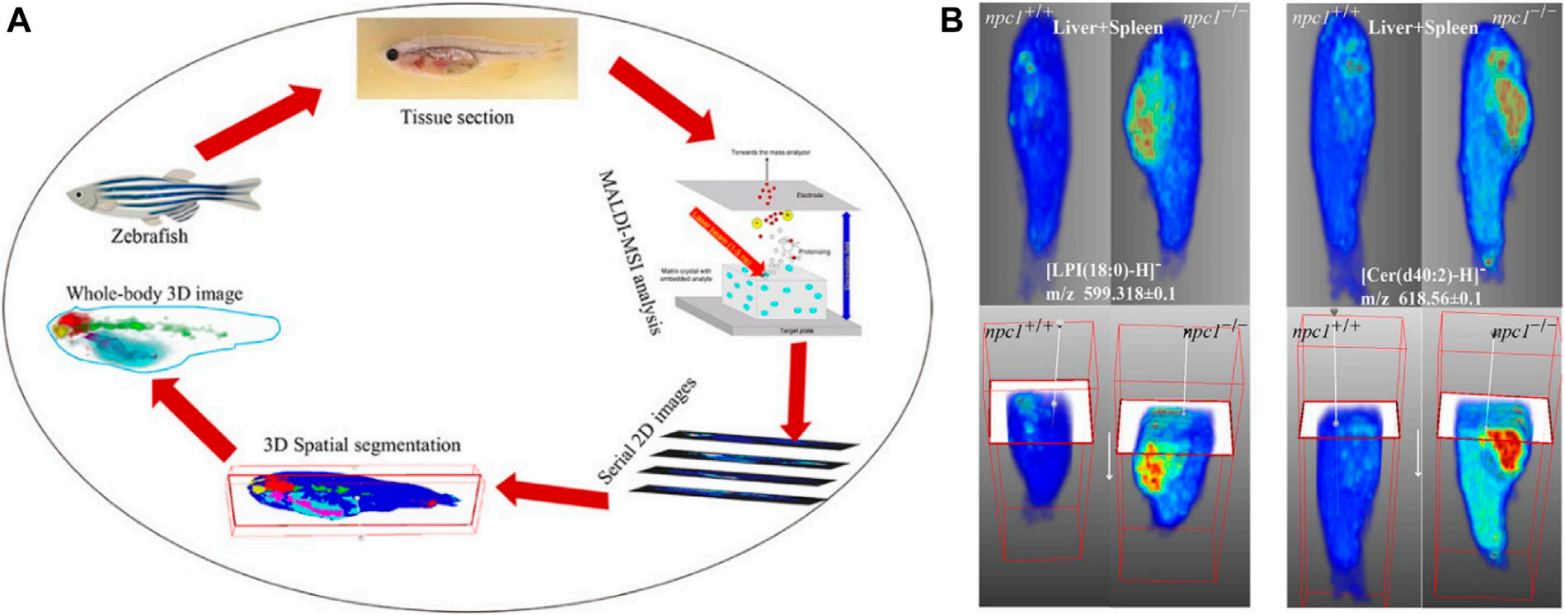
Three dimensional (3D) reconstruction of zebrafish by MALDI-MSI (Liang et al., 2021). **(A)** The workflow of the 3D MALDI-MSI method. **(B)** Spatial distributions of Cer (d34:1) and Cer (d37:1) in fish brain.

A 3D MALDI-MSI method (Figure 10) was applied for whole-body analysis of zebrafish and was used to identify altered lipids and map their spatial distribution within zebrafish model Niemann–Pick disease type C1 (NPC1), a neurovisceral lipid storage disorder. The constructed 3D model of fish provided comprehensive information on the 3D distribution of lipids and allowed direct correlation between these lipids and fish organs. The results showed that compared with the wild type, some sphingolipids and phospholipids in the brain, spinal cord, intestines, and liver–spleen region of fish with NPC1 gene mutation had significant changes and showed different localization patterns. This 3D MALDI-MSI method can provide a global picture of lipid changes in different organs and functional systems (Liang et al., 2021).

To investigate the possible role of epididymis in the complex maturation of sperm, MALDI-MSI investigated the precise location of lipid metabolites in the rat epididymis, mainly detecting phosphatidylcholines, sphingolipids, glycerophosphates, triacylglycerols, plasmalogens, phosphatidylethanolamines, and lysophosphatidylcholines. During epididymal maturation, the number of sphingolipids and plasmalogens increased, while the proportion of triacylglycerols decreased from caput to cauda. Molecules belonging to the same family may have very different positions on the epididymis. A 3D model of the epididymis head was also reconstructed by 3D MALDI-MSI, which can be used to obtain localization information of specific analytes in the entire tissue. This work opens a new perspective on the role of lipid metabolism in sperm maturation when it moves through the epididymis (Lagarrigue et al., 2020).

### 6.2 Three-Dimensional Mapping Together Light Sheet Fluorescence Microscopy

Light sheet fluorescence microscopy (LSFM) of optically cleared biological tissue samples has developed rapidly in the past decade and has become a powerful tool for 3D histomorphological analysis applied to various life sciences (Hillman et al., 2019; Ueda et al., 2020).

**FIGURE 11:**
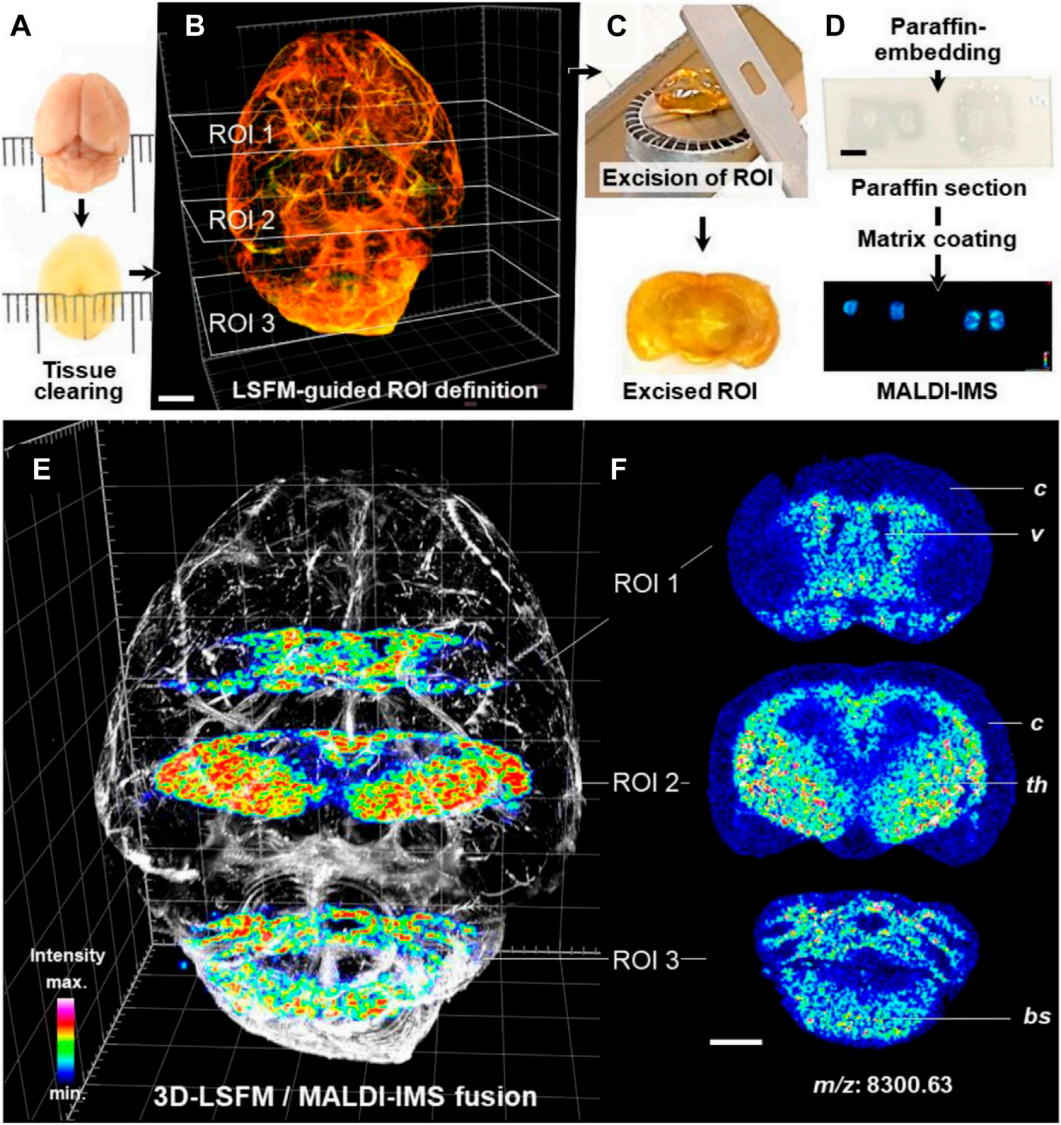
3D-LSFM-guided MALDI-MSI in an optically cleared mouse brain (Blutke et al., 2020). **(A–D)** Sequence of tissue-processing steps. **(E)** Fused image of the 3D-LSFM reconstruction of the cleared brain and MALDI-MS images of guanine nucleotide-binding protein subunit gamma-3 (GNG3, *m/z*: 8,300.63). **(F)** MALDI-MS images of GNG3. The spatial distribution of GNG3. Distinct brain structures are indicated for orientation: cerebral cortex (*c*), ventricles (*v*), thalamus (*th*), brain stem (*bs*).

LSFM of cleared brain tissue samples could be combined with MALDI-MSI for protein detection and quantification (Figure 11). Fresh dissected murine brain tissue and archived FFPE human brain tissue were cleared. Regions of interest of tissue defined by LSFM were paraffin embedded and sectioned. The sections were then subjected to MALDI-TOF-MSI in mass ranges between 0.8 and 4 kDa (human) or 2.5–25 kDa (mouse) with a lateral resolution of 50 µm. The protein and peptide characteristics corresponding to the obtained MALDI-MSI spectra were determined by parallel LC-MS/MS analysis. MALDI-MSI will be of great value in combination with qualitative and quantitative morphological analysis of complex 3D tissue structures (Blutke et al., 2020).

### 6.3 Three-Dimensional Imaging Host–Microbe Interactions by Combining Mass Spectrometry Imaging and X-Ray Tomography

**FIGURE 12:**
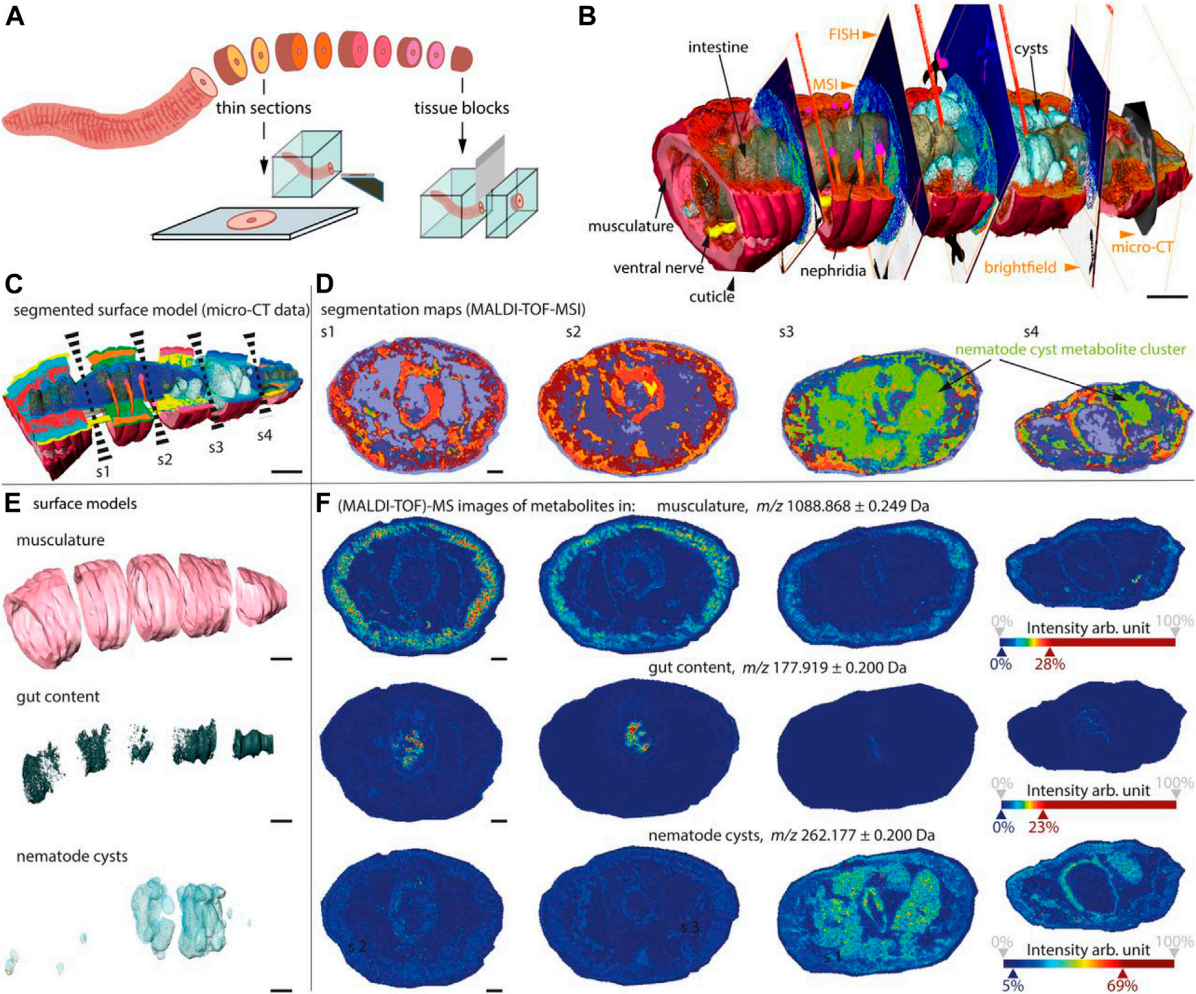
CHEMHIST revealed organ-specific chemistry in the posterior segments of an earthworm (Geier et al., 2021).

As is known to all, metabolites mediate most interkingdom symbioses. However, determining the metabolites of each member of the biological interaction remains a huge challenge. Recently, a chemo-histo-tomography (CHEMHIST) method (Geier et al., 2021) has been developed to link histological changes with metabolites by combining MSI and x-ray tomography (micro-CT) to correlate metabolite distribution with 3D histology of the same animal (Figure 12), down to submicrometer resolutions. This method is compatible with tissue-specific DNA sequencing and fluorescence *in situ* hybridization and can be used for taxonomic identification and localization of relevant microorganisms. These results revealed the physical and chemical interactions of an earthworm from its natural habitat with bacteria and parasitic nematodes in its tissues. Combined MSI and micro-CT, advances in chemical and structural *in situ* imaging will drive the study of metabolic interactions in symbiotic systems (Geier et al., 2021).

## 7 Future Directions

MALDI-MSI is now a promising tool for rapid and robust molecule-specific MS imaging of biological tissue sections at a broad range of length scales, ranging from the subcellular level to whole-body tissue sections. As the sample applied in MALDI-MSI varied, it is critical to standardize sample preparation, storage protocols, and data acquisition (Dihazi et al., 2013). Flatley et al. pointed out that many researches were of highly contradictory results and existing repeat mistakes for lack of standard operating protocols (SOPs) (Flatley et al., 2014).

Speed, specificity, spatial resolution, and sensitivity, the “4S-criteia for performance,” is still suitable for further MALDI-MSI (Schulz et al., 2019). Improving the MSI resolution to single-cell level and even subcellular level will make it more suitable and useful for deeper biological discovery. It is for sure that MALDI reduces the disturbance to the cell. However, when it comes to single-cell level, the complexity of sample preparation of clinical samples may cause analyte delocalization. From an instrumental side, laser and detector technologies must be developed to meet the need of speed and accuracy in high-throughput analysis. Besides, advanced computational solutions must be developed to handle the problems of large-scale data, processing, integration, and storage (Scupakova et al., 2020).

A significant challenge for most MSI is the failure to distinguish isomers, which may cause the misinterpretation of location and function of isomers, due to the lack of chromatography step. For example, fructose has been shown to contribute to the Warburg effect and cancer growth (Port et al., 2012; Nakagawa et al., 2020). However, it is difficult to image fructose using the ordinary MSI method due to the interference of its common isomer—glucose, which plays a minor role of energy source for cancer growth, different from fructose (Nakagawa et al., 2020). This limitation could be overcome by coupling MSI with approaches capable of resolving the isobaric molecules, such as tandem mass spectrometry (MS/MS) (Zhan et al., 2021), trapped ion mobility spectrometry (TIMS) (Sans et al., 2018; Spraggins et al., 2019). Recent efforts in coupling special reaction to tandem mass spectrometry imaging have succeeded in distinguish lipid isomers based on strategy for identifying C=C bond positions, such as on-tissue Paterno–Buchi reaction (Bednarik et al., 2018; Waldchen et al., 2019), ozone-induced dissociation (OzID) (Paine et al., 2018; Claes et al., 2021; Young et al., 2021), and online photochemical derivatization (Unsihuay et al., 2021b). There is also a strategy utilizing structure-specific derivatization methods to modify one of the isomers to separate isomers before sampling, which is rarely available.

**FIGURE 13:**
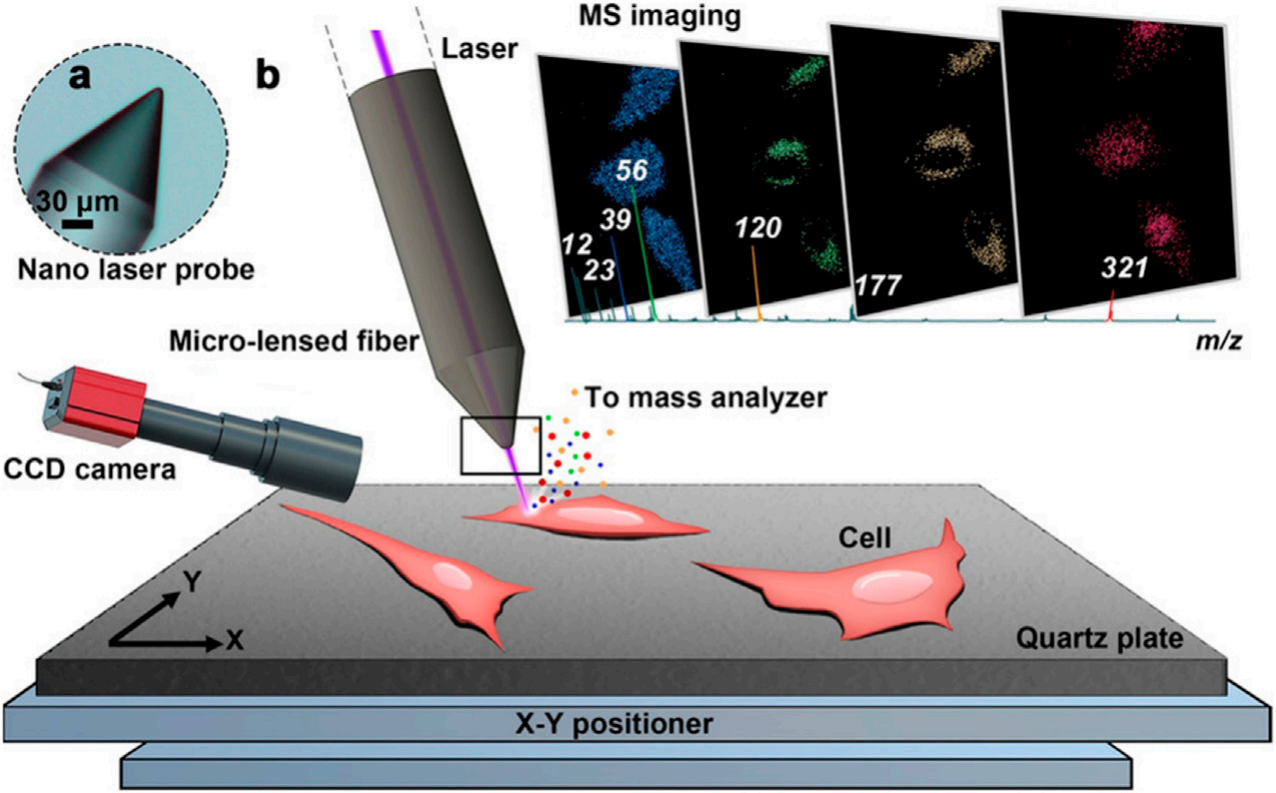
Nano laser probe-based MSI system. **(A)** A microscope photograph of the tip of the nano laser probe (NLP). **(B)** Diagram of the ion source and MSI process (Meng et al., 2020).

MALDI and SIMS are important ionizations applied in subcellular-level MSI (Kallback et al., 2012). Compared with MALDI-MSI, SIMS-MSI can obtain higher spatial resolution images directly from biological tissue. Nowadays, nanoSIMS with a resolution of nm level has been applied in quantitation of subcellular protein (Vreja et al., 2015), lipid (Jiang et al., 2014), neurotransmitter (Lovric et al., 2017), drug (Jiang et al., 2017), and even DNA (Steinhauser et al., 2012) distribution in a single cell, but recent achievements in MALDI-MSI show that using MALDI as ionization can approach nm-level resolution. The use of t-MALDI-2 MSI system achieved a high pixel size of 600 nm with brain tissue (Niehaus et al., 2019). Besides, as shown in Figure 13, the resolution of recent nano laser probe-based MSI systems can be down to the nm level (subcellular level) by introducing desorption laser with a micro-lensed fiber, proving a great development in MALDI-MSI (Meng et al., 2020). Considering the lack of selectivity of samples of MALDI ionization (Susniak et al., 2020) and the ability to detect DNA (Kirpekar et al., 1999; Ehrich et al., 2005), we believe that MALDI will become more and more important in MSI.

## 8 General Marks and Conclusions

MSI is a powerful analytical technique that cannot only detect qualitatively and determine quantitatively hundreds and thousands of a large variety of natural and synthetic compounds, such as lipids, amino acids, metabolites, peptides, proteins, DNA, RNA, and even SARS-CoV-2 virus (Nachtigall et al., 2020), but also can map simultaneously spatial locations of these detected molecules. Therefore, there are increasing trends to apply MSI where required to know the relative abundance and spatial distribution of the molecules. Compared with other MSI techniques, such as SIMS and DESI, MALDI-MSI is a very simple, economic, and reliable technique. It does not need assisting solvents/gas jet or special ion beam, but only requires a suitable matrix on the sample plate and a pulsed laser beam for ionizing the targeted molecules. Laser desorption ionization together with assisted matrix made MALDI more practical, salt resistant, and sensible than other methods. In addition, the majority of ions ionized by MALDI are singly protonated; thus, the molecular weight could be common directly read, and less MS noises besides matrix signals have been detected. With the developments of organic matrix-free nano matrices such as GO, nano gold, titanium oxide and nanowires, and on-tissue or on-cell chemical derivation, MALDI-MSI will provide high sensitivity. Furthermore, MALDI ion resources can be easily coupled with high-resolution (resolving power) mass analyzers, such as TOF, FT-ICR, and Orbitrap, which provide high mass accuracy for MSI targets. During the recent years, with the developments of some new MALDI ion sources, such as atmosphere pressure AP-MALDI (Kompauer et al., 2017), AP-SMALDI (Vegvari et al., 2017; Bhandari et al., 2018; Bredehoeft et al., 2019; Kadesch et al., 2020; Mokosch et al., 2021; Mueller et al., 2021; Righetti et al., 2021), MALDI-2 (Soltwisch et al., 2015), transmission MALDI-2 (Niehaus et al., 2019), and nano laser probed-based laser desorption ionization (Meng et al., 2020), the lateral resolution of MALDI-MSI will possibly achieve nm or µm level for single cell and even subcellular scale imaging. Finally, the scanning speed of MALDI-MSI largely depends on the speed of MS detection, spectra recording and data processing. With the developments of MSI instruments and artificial intelligence for big data, the MALDI-MSI will achieve high-speed scanning and rapid analyses. Therefore, further high-resolution MALDI-MSI will be applied to wide fields from single cells, tissues, to 3D organisms for molecular understanding of life and other human-related fields.
